# epiTCR: a highly sensitive predictor for TCR–peptide binding

**DOI:** 10.1093/bioinformatics/btad284

**Published:** 2023-04-24

**Authors:** My-Diem Nguyen Pham, Thanh-Nhan Nguyen, Le Son Tran, Que-Tran Bui Nguyen, Thien-Phuc Hoang Nguyen, Thi Mong Quynh Pham, Hoai-Nghia Nguyen, Hoa Giang, Minh-Duy Phan, Vy Nguyen

**Affiliations:** Medical Genetics Institute, Ho Chi Minh City, Vietnam; Medical Genetics Institute, Ho Chi Minh City, Vietnam; Medical Genetics Institute, Ho Chi Minh City, Vietnam; NexCalibur Therapeutics, Wilmington, DE, United States; Medical Genetics Institute, Ho Chi Minh City, Vietnam; Medical Genetics Institute, Ho Chi Minh City, Vietnam; Medical Genetics Institute, Ho Chi Minh City, Vietnam; NexCalibur Therapeutics, Wilmington, DE, United States; University of Medicine & Pharmacy, Ho Chi Minh City, Vietnam; Medical Genetics Institute, Ho Chi Minh City, Vietnam; NexCalibur Therapeutics, Wilmington, DE, United States; Medical Genetics Institute, Ho Chi Minh City, Vietnam; NexCalibur Therapeutics, Wilmington, DE, United States; Medical Genetics Institute, Ho Chi Minh City, Vietnam

## Abstract

**Motivation:**

Predicting the binding between T-cell receptor (TCR) and peptide presented by human leucocyte antigen molecule is a highly challenging task and a key bottleneck in the development of immunotherapy. Existing prediction tools, despite exhibiting good performance on the datasets they were built with, suffer from low true positive rates when used to predict epitopes capable of eliciting T-cell responses in patients. Therefore, an improved tool for TCR–peptide prediction built upon a large dataset combining existing publicly available data is still needed.

**Results:**

We collected data from five public databases (IEDB, TBAdb, VDJdb, McPAS-TCR, and 10X) to form a dataset of >3 million TCR–peptide pairs, 3.27% of which were binding interactions. We proposed epiTCR, a Random Forest-based method dedicated to predicting the TCR–peptide interactions. epiTCR used simple input of TCR CDR3β sequences and antigen sequences, which are encoded by flattened BLOSUM62. epiTCR performed with area under the curve (0.98) and higher sensitivity (0.94) than other existing tools (NetTCR, Imrex, ATM-TCR, and pMTnet), while maintaining comparable prediction specificity (0.9). We identified seven epitopes that contributed to 98.67% of false positives predicted by epiTCR and exerted similar effects on other tools. We also demonstrated a considerable influence of peptide sequences on prediction, highlighting the need for more diverse peptides in a more balanced dataset. In conclusion, epiTCR is among the most well-performing tools, thanks to the use of combined data from public sources and its use will contribute to the quest in identifying neoantigens for precision cancer immunotherapy.

**Availability and implementation:**

epiTCR is available on GitHub (https://github.com/ddiem-ri-4D/epiTCR).

## 1 Introduction

In human immune system, cytotoxicity T cells can specifically recognize and kill cancer cells, and other cells that are infected or damaged. It is activated by the binding of CD8+ T-cell receptor (TCR) with the antigen presented via the class I major histocompatibility complex (MHC-I). If one can reliably predict which cancer-derived antigen can elicit CD8+ T-cell response, such antigen can be used in immunotherapy to boost CD8+ T-cell activity and precisely target cancer cells that present the same antigen. Therefore, the prediction of TCR–peptide binding has become an important study objective, the solution of which can lead to breakthroughs in immunotherapy. Many computational approaches have been proposed to predict TCR–peptide binding at large scales. They are diverse in terms of required information, algorithms, and datasets.

While a short peptide sequence is the required input for TCR–peptide binding prediction, the paired TCR components are quite different among predicting tools. A large number of tools [NetTCR (Montemurro et al. 2021), ATM-TCR (Cai et al. 2022), pMTnet (Lu et al. 2021), ImRex (Moris et al. 2021), ERGO-I (Springer et al. 2020), TCRGP (Jokinen et al. 2021), and TITAN (Weber et al. 2021)] only takes the sequences of complementarily determining region-3 beta (CDR3β) because this region is mainly responsible for peptide binding. Some tools [NetTCR (Montemurro et al. 2021), ERGO-II (Springer et al. 2021), TCRGP (Jokinen et al. 2021), TCRAI (Zhang et al. 2021b)] use additional CDR3α sequences because they have been proven to support the CDR3β–peptide binding. A few other tools [ERGO-II (Springer et al. 2021), TCRAI (Zhang et al. 2021b), TCRex (Gielis et al. 2018, 2019)] also consider the V, D, and J segments, with the participation of MHC sequences to further reveal the TCR–pMHC binding complexes. However, the more data used as input, the wider the need for diverse annotated binding complexes as ground truth for both binding and non-binding interactions. Some prediction tools [ERGO-II (Springer et al. 2021), TCRGP (Jokinen et al. 2021)] use randomly generated data as non-binding controls, which risks dampening the prediction’s accuracy.

The core algorithm is also different among computational approaches. Early approaches use machine-learning strategies. Indeed, TCRGP (Jokinen et al. 2021) learns Bayesian non-parametric models from covariance matrices of TCR sequences, and TCRex (Gielis et al. 2018, 2019) applies a random forest-learning model for each peptide in the training set. TCRdist (Dash et al. 2017) clusters similar TCRs based on the distance between two TCR, then assigns the new TCR binding to peptides bound by other TCRs in the same cluster. RACER (Lin et al. 2021) optimizes an energy pairwise model to distinguish strong binding from weak bindings, but this approach is limited to known structures to infer the binding energy between TCR and peptide. Many recent approaches employ deep learning models for binding classification. In particular, NetTCR (Montemurro et al. 2021), DeepTCR (Sidhom et al. 2021), and ImRex (Moris et al. 2021) use convolutional neural networks (CNN) to extract important binding patterns. ATM-TCR (Cai et al. 2022) and TITAN uses attention-based neural networks, while ERGO-I (Springer et al. 2020) and pMTnet (Lu et al. 2021) embeds TCR and peptide by long short-term memory (LSTM) and autoencoder (AE), followed by fully connected neural networks for pattern learning. In general, all currently available tools offer prediction with high specificity but low sensitivity. Furthermore, more complex learning structures require specific computational resources, i.e. GPU, which needs more investigation from researchers.

Most available learning models used a small number of datasets for training. Indeed, NetTCR (Montemurro et al. 2021) model was trained on data from The Immune Epitope Database (IEDB) (Vita et al. 2019) and VDJdb (Shugay et al. 2018; Bagaev et al. 2020). ERGO-II (Springer et al. 2021) and TCRex (Gielis et al. 2018, 2019) used VDJdb and McPAS-TCR (Tickotsky et al. 2017). TCRGP (Jokinen et al. 2021) trained model from VDJdb (Shugay et al. 2018; Bagaev et al. 2020) and Dash et al*.* (2017) data published in TCRdist. ImRex (Moris et al. 2021) and TCRdist (Dash et al. 2017) retrieved only VDJdb (Shugay et al. 2018; Bagaev et al. 2020) for training, while DeepTCR (Sidhom et al. 2021) and TCRAI (Zhang et al. 2021b) trained from only 10X (https://www.technologynetworks.com/immunology/application-notes/a-new-way-of-exploring-immunity-linking-highly-multiplexed-antigen-recognition-to-immune-repertoire-332v554). ATM-TCR (Cai et al. 2022) and ERGO-I (Springer et al. 2020) collected all mentioned databases as binding data. pMTnet is a rare tool that collected training data from 10 datasets (Lu et al. 2021), but the number of data points used for training was surprisingly small (over 32 000 binding complexes) (https://github.com/tianshilu/pMTnet). A common training strategy shows the training on one dataset and the model testing on another distinct dataset [f.e., NetTCR (Montemurro et al. 2021)] tested on the MIRA dataset (Klinger et al. 2015), while ImRex and DeepTCR tested on McPAS-TCR). This strategy was possibly preferred to assure the neutrality of testing. However, an important drawback of such a strategy is to limit the model learning within the peptides reported in the training dataset. Additionally, it is worth discussing the choice of non-binding complexes. While 10X validated data was reasonably chosen by NetTCR and pMTnet, many other classification tools randomly selected non-binding data by switching different unreported TCR–peptide pairs from validated binding pairs (ERGO-I, ERGO-II, TITAN). These risks introducing false non-bindings into the ground truth. Nevertheless, ImRex (Moris et al. 2021) retrieved data from Dean et al*.* (2015), which consists of healthy donors, and assumed that TCR from healthy donors would not bind to any peptide sequences. In fact, validated data from 10X showed that a small number of TCR from healthy donors still bind to some of these peptides.

All important aspects above highlight the reasons that impede the performance of all existing TCR–peptide predicting applications. Here we present epiTCR, a Random Forest-based model to predict the binding between provided TCR and peptide sequences. Having been trained on a large data collection from five public TCR–peptide binding databases (Supplementary Table S1), epiTCR gives prediction with high sensitivity while preserving good specificity. The model uses only CDR3β as TCR input data and reveals seven epitopes that are challenging for prediction.

## 2 Materials and methods

### 2.1 Data collection

**Figure 1.**
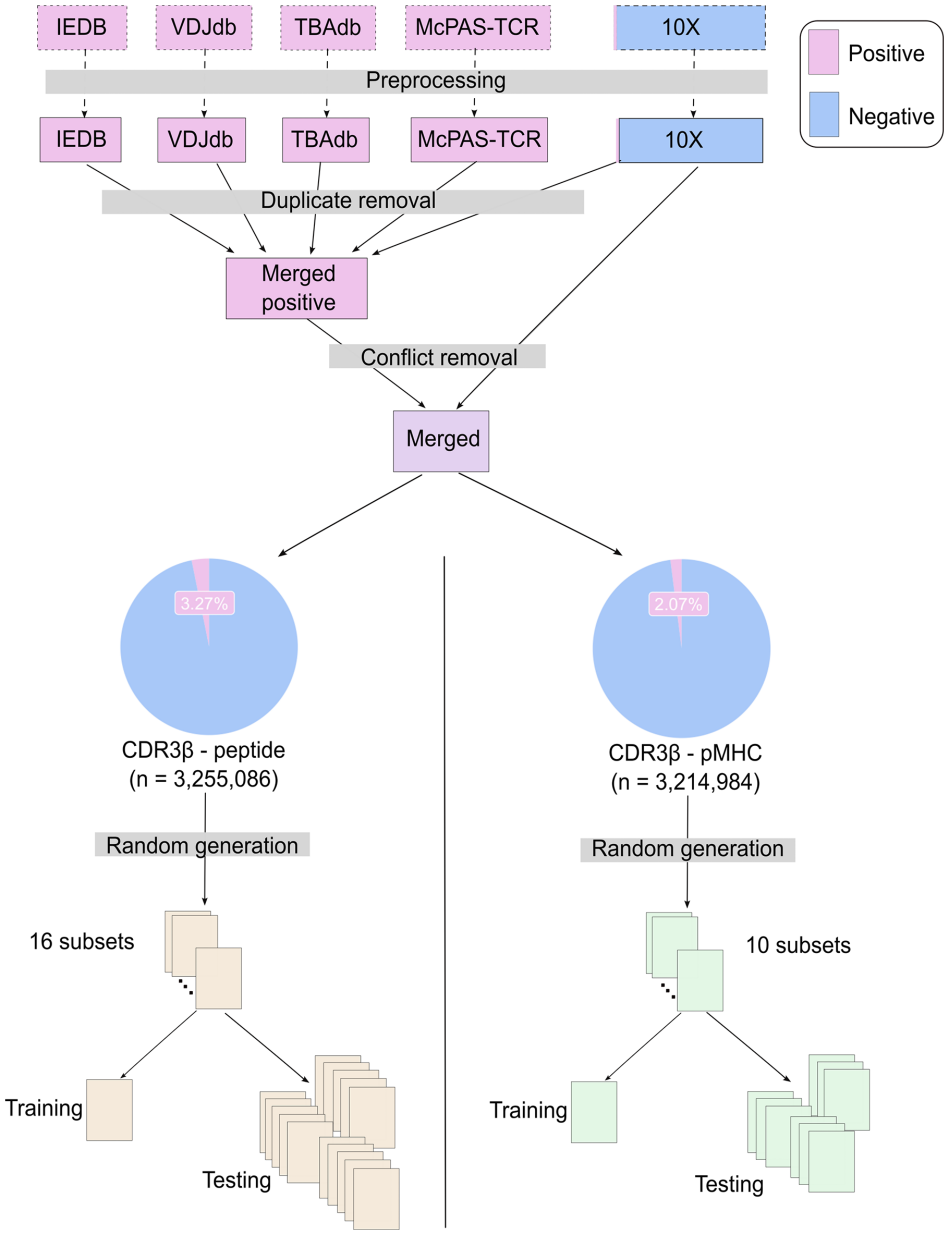
Diagram illustrating the collection of data from five publicly available databases, the data processing steps, and the organizing data into training and testing sets for CDR3β–peptide and CDR3β–pMHC input data. A 3.27% and 2.07% of the observations are binding data, in the CDR3β–peptide pairs and CDR3β–pMHC complexes, respectively.

To maximize the amount of data we can use to train epiTCR, we gathered data from five public datasets [TBAdb (Zhang et al. 2020), VDJdb (Shugay et al. 2018; Bagaev et al. 2020), McPAS-TCR (Tickotsky et al. 2017), IEDB (Vita et al. 2019), and 10X], processed them to remove duplicates and conflicts before merging into a unified dataset which was subsequently split for training and testing (Fig. 1; Supplementary Table S1). VDJdb (Shugay et al. 2018; Bagaev et al. 2020), McPAS-TCR (Tickotsky et al. 2017), IEDB (Vita et al. 2019), and TBAdb (Zhang et al. 2020) are records of validated TCR–peptide binding pairs that have been curated manually. Particularly, TBAdb contains many interactions retrieved from Asian patients, which is relatively rare in other databases and have not been used to train many existing tools (except pMTnet). The dataset from 10X contains mostly validated non-binding data, but a small number of binding interactions in this dataset also help enrich the binding curated information. After that, a non-redundant and non-conflicted list of binding complexes across all datasets was randomly sampled to form peptide-diverse training and model testing. Importantly, we noticed that the data can be divided into those with corresponding MHC information and those without. We decided to build epiTCR for both cases of input data. Therefore, 10 subsets with MHC (70 000–75 000 records each) and 16 subsets without MHC complexes (70 000–75 000 records each) were formed. This led to over 7000 distinct binding pairs in each subset, a comparable number with the data used in NetTCR (ranging from 1000 to 9000 pairs). One subset was used for model training, and the remaining subsets were used for testing.

### 2.2 Model training

**Figure 2.**
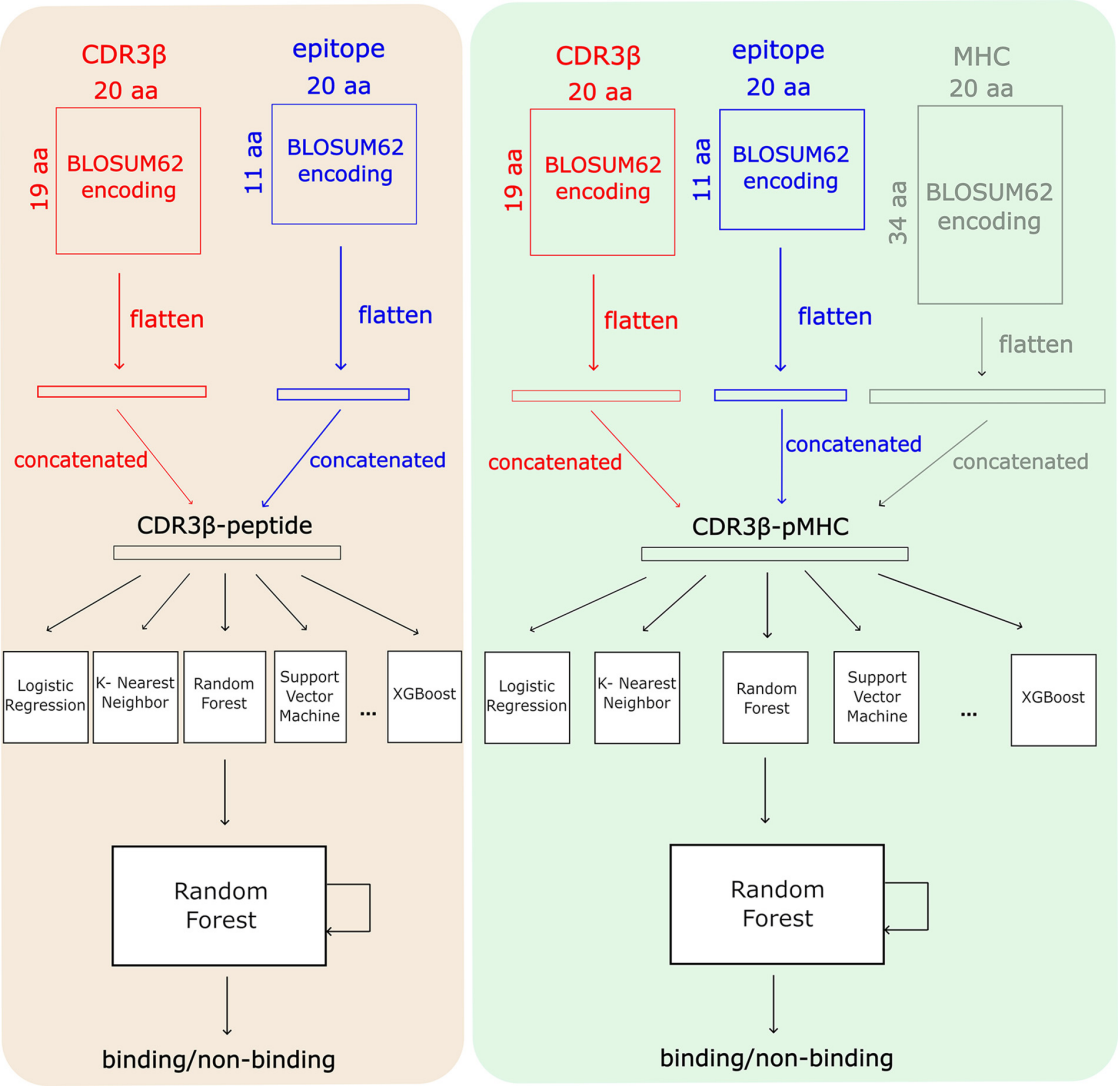
Overview of training and predicting processes in two modes, epiTCR and epiTCR–pMHC.

TCR–peptide binding classification is a challenging prediction problem due in part to the heavily imbalanced data. Complex learning structures, such as neural networks, may not be the optimal choices for such extremely imbalanced data. On the other hand, machine learning with more training strategies (either rich learning data or feature selection methods), can give better solutions. epiTCR is resulting from our attempts on using machine learning to solve the classification problem using a large amount of publicly available data.epiTCR was developed in python using scikit-learn (Pedregosa et al. 2012) version 1.1.2 inbuilt Random Forest for model training and data prediction. CDR3β sequences of 8–19 amino acids were provided as TCR input, and peptide sequences of 8–11 amino acids were given as the corresponding peptide. TCR and peptide sequences were then encoded individually using BLOSUM62 (Henikoff and Henikoff 1992) encoding for 20 amino acids, while zero padding was applied for short sequences, leading to a matrix of 20 × 19 and 20 × 11 for each TCR and peptide sequence, respectively. The matrices were then flattened into vectors and concatenated to form a vector of 600 features (Fig. 2). This features vector was then provided as input for the training. Binding and non-binding information of the TCR–peptide pairs was also encoded as 1 and 0, respectively, to be supplied to the training as labels. We trained the classification models using the Random Forest based-approach, the best performed among machine learning models evaluated through five-fold cross validation on the training set (Supplementary Table S2). In the prediction phase, new TCR–peptide binding pairs in question were also encoded, represented, and provided to the learned model. The classification phase produced the prediction with probability indicating the reliability of the binding interaction. Here we used the default prediction probability threshold of 0.5.

To evaluate the role of antigen-presenting molecules (MHC) in the prediction of TCR–peptide binding, epiTCR was also extended to an epiTCR–pMHC running mode where peptide-presenting MHC in human can also contribute to the training. The provided MHC information in form of HLA nomenclature is then converted into IMGT annotated peptide sequences (Robinson et al. 2000, 2020). Consequently, long HLA sequences were converted to short 34-amino acid long pseudo-sequence following the method proposed in NetMHCpan (Nielsen et al. 2007). Using the same data representation method as for the TCR and peptide, the flattened vector for HLA is 680 elements, leading to a total of 1280 features for the concatenated input vector. Since many methods have been proposed for pMHC binding prediction [NetMHCpan (Hoof et al. 2009; Jurtz et al. 2017; Nielsen and Andreatta 2016; Nielsen et al. 2007; Reynisson et al. 2020), PRIME (Schmidt et al. 2021), and IEDB immunogenicity predictor (Calis et al. 2013), ForestMHC (Boehm *et al.* 2019)], epiTCR does not reconsider the pMHC interaction but assumes that the binding complexes are confirmed.

## 3 Results

### 3.1 Random Forest was the model of choice for prediction of TCR–peptide interactions

This project was started with data collected from five public datasets [TBAdb (Zhang et al. 2020), VDJdb (Shugay et al. 2018; Bagaev et al. 2020), McPAS-TCR (Tickotsky et al. 2017), IEDB (Vita et al. 2019), and 10X]. Through a series of pre-processing steps (see Section 2 and Supplementary Methods), the final dataset comprised of 3 255 086 CDR3β–peptide combinations (including 3 148 510 non-binding combinations), which were randomly split into 16 subsets (70 000–75 000 records each) for model training and testing (Fig. 1). Multiple classification models including SVM (with linear, polynomial, sigmoid, and RBF kernels), k-nearest neighbors, Random Forest, XGBoost, Linear Regression (with lbfgs, liblinear, sag, newton-cg solvers), and Gaussian Naive Bayes were trained. Through 5-fold cross-validation on the training set, Random Forest achieved the highest accuracy, followed by polynomial SVM and linear SVM (Table 1; Supplementary Table S2). Additionally, Random Forest exhibited the shortest running time (fit time) among the top five best performing models. We therefore chose Random Forest model for further optimization and comparison with other available tools.

**Table 1.** Performance comparison of machine-learning models using 5-fold cross-validation

Models	Mean validation accuracy	Mean validation AUC	Mean fit time (in seconds)
Random Forest	0.920	0.980	4.804
SVM—[kernel: poly]	0.919	0.964	22.878
SVM—[kernel: linear]	0.918	0.969	166.039
XGBoost	0.917	0.979	46.683
SVM—[kernel: rbf]	0.916	0.961	29.170
Logistic Regression [solver: liblinear]	0.914	0.975	18.610
Logistic Regression [solver: newton-cg]	0.914	0.975	8.176
Logistic Regression [solver: lbfgs]	0.911	0.973	0.184
KNN—[n_neighbors: 3]	0.888	0.942	0.069
KNN—[n_neighbors: 2]	0.862	0.925	0.060
Bernoulli	0.761	0.847	0.090
SVM—[kernel: sigmoid]	0.656	0.690	31.230

The mean values have been rounded to three decimal places.

### 3.2 epiTCR outperformed existing tools in terms of AUC and sensitivity

We next compared the performance of Random Forest-based epiTCR with four existing network-based TCR–peptide prediction tools: NetTCR (Montemurro et al. 2021), ImRex (Moris et al. 2021), ATM-TCR (Cai et al. 2022), and pMTnet (Lu et al. 2021). While the first three tools require minimum TCR input as CDR3β sequences, pMTnet (Lu et al. 2021) offers binding prediction with the participation of MHC data. These tools also vary in data representations and learning algorithms. NetTCR (Montemurro et al. 2021) uses BLOSUM50 matrix (Henikoff and Henikoff 1992) for data representation, ImRex (Moris et al. 2021) encodes the sequences using physicochemical properties (mass, hydrophobicity, hydrophilicity, and isoelectric point), and pMTnet (Lu et al. 2021) represents the pMHC with BLOSUM50, and CDR3β with five Atchley factors (Atchley et al. 2005) learned from AE. ATM-TCR (Cai et al. 2022) is the only tool that uses one hot encoding for data representation. In contrast to epiTCR which used a simple Random Forest model, all four tools use neural networks but with some differences in their network structures. ATM-TCR integrates scaled dot products as self-attention training to extract important features, while NetTCR and ImRex learn important patterns by gradually applying multiple filters different in size. pMTnet has the most complicated training structure where pMHC binding was first evaluated by LSTM, then integrated with the AE-learnt CDR3β in the deep learning network. Among these tools, NetTCR (Montemurro et al. 2021) and ATM-TCR (Cai et al. 2022) were benchmarked twice with the authors’ default models and retrained models using the same training set as epiTCR. These tools were chosen for benchmarking based on similar input requirements, and the availability of reproducible running codes.

**Figure 3.**
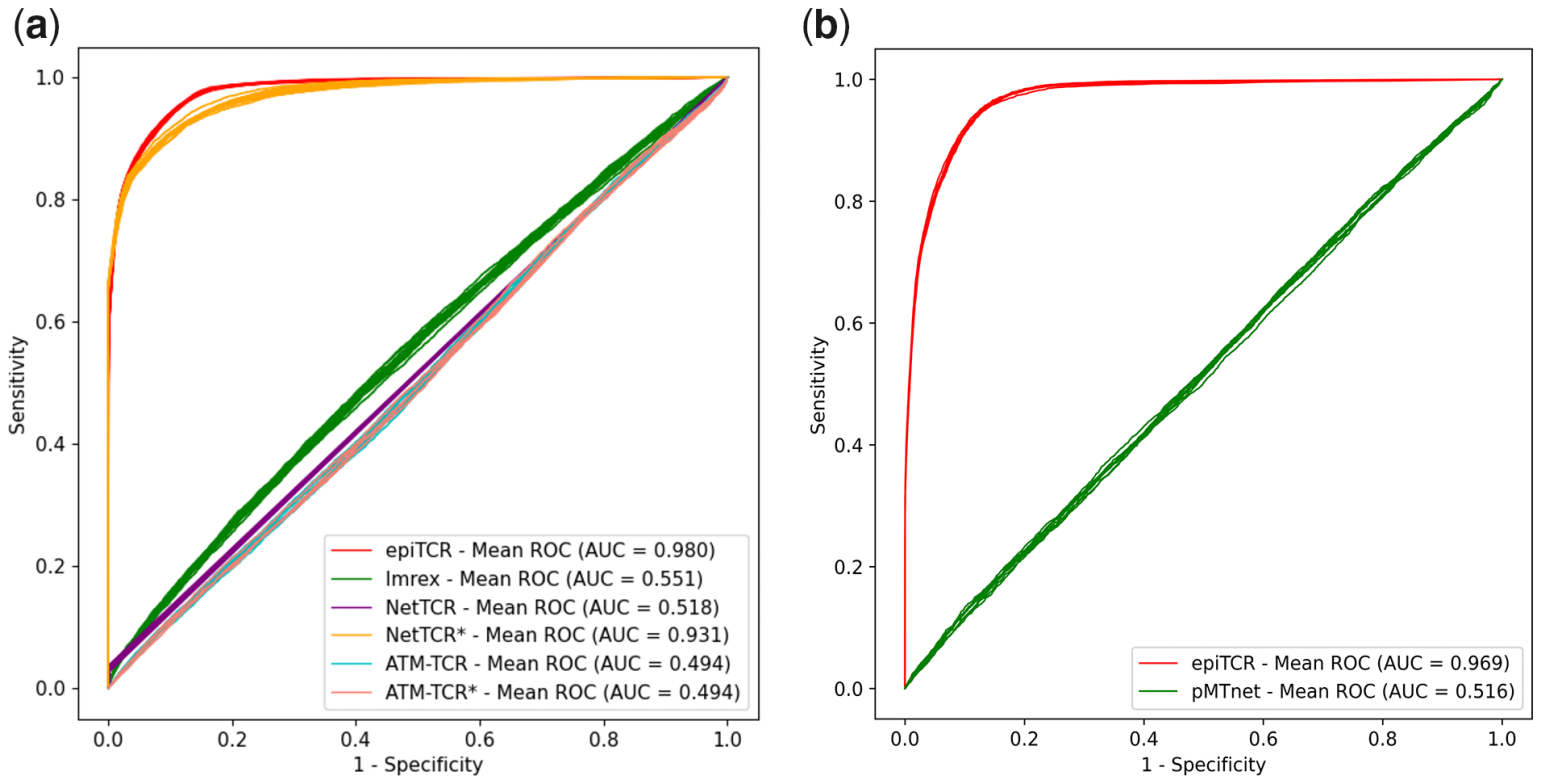
The performance of epiTCR, NetTCR, Imrex and ATM–TCR, and pMTnet in two predicting modes: (a) TCR–peptide and (b) TCR–pMHC binding, across 15 test sets and 9 test sets, respectively. ROC curves for all tests were reported with the mean AUC score indicated as texts. An asterisk (*) after a tool name indicates a retrained model.

The first benchmark without the participation of MHC was tested on 15 subsets randomly selected, distinctive from each other and from the training set (Fig. 1). This means no single TCR–peptide pair is found in multiple sets. All binding pairs were exhaustively used, while the non-binding observations were chosen at 10 times the number of binding ones. The performance was accessed by four metrics: AUC, accuracy, sensitivity, and specificity. The nature of the binding versus non-binding prediction is an imbalance two-group classification in which the number of non-binding pair is significantly larger than that of binding pair. Therefore, the benchmark on the AUC score provides the most neutral comparison. The result showed that ImRex and two models of ATM-TCR (original and retrained) had low performance in all benchmarked metrics with the AUC ranging from 0.49 to 0.55 (Fig. 3a and Supplementary Fig. S2). epiTCR outperformed all other benchmarked tools with the mean AUC at 0.98 and the sensitivity of 0.94. At most of the points on the ROC curve, epiTCR produced higher sensitivity, whilst the retrained NetTCR model performed with slightly higher accuracy and specificity at certain probability cutoffs, particularly in the default probability cutoff at 0.5 (Fig. 3a). The original model of NetTCR, however, suffered from poor sensitivity. Altogether, epiTCR was the only tool having the ability to capture a large number of binding pairs while maintaining a reasonably high specificity (0.9).

To fully characterize epiTCR performance, we stratified our data into different groups depending on the sources of peptides (virus or human) or level of binding validation (antigen-specific validated data and antigen-specific association data). We then examined epiTCR performance in predicting CDR3β–peptide binding in these groups of data. Indeed, the AUC of epiTCR prediction was relatively robust when we predicted TCR binding with different pathogen sources (Supplementary Fig. S4) and data validation (Supplementary Fig. S5).

Our second benchmark evaluated the performance of epiTCR in predicting the TCR–pMHC binding compared to pMTnet. Limited by the amount of known labeled data, only nine distinct subsets were available for testing (Fig. 1). Consistent with the first benchmark without MHC, this result showed higher performance of epiTCR in AUC (0.97 compared with 0.51) and sensitivity (0.94 at prediction cutoff 0.5 for epiTCR versus 0.03 at ranking cutoff 0.02 for pMTnet), equal performance of epiTCR in accuracy (0.89), and slightly lower specificity compared with pMTnet (0.88 compared with 0.98, with prediction cutoff and ranking cutoff at 0.5 and 0.02, respectively) (Fig. 3b and Supplementary Fig. S6). pMTnet had similar performance with other models in the first benchmark (except retrained NetTCR) that they both have remarkably high specificity, hence leading to slightly higher prediction accuracy. However, pMTnet showed extremely low sensitivity (0.03–0.04) at the ranking cutoff of 0.02.

Overall, epiTCR showed better performance than all benchmarked tools in terms of AUC and sensitivity while maintaining good specificity. This implies that epiTCR is the only tool that provides a harmonious trade-off between sensitivity and specificity. Among the two prediction models provided by epiTCR, the MHC-participating model has slightly lower performance in terms of AUC and specificity. This might be because MHC is not antigen-specific but patient-driven, hence the supportive role of MHC in CDR3β–peptide binding was not recognized by epiTCR. In another aspect, the competitive performance given by the NetTCR retrained model suggests a possibility that a model's performance can be improved with additional data. This information is important since more and more labeled binding data are becoming publicly available.

### 3.3 epiTCR revealed epitopes challenging for binding prediction

**Figure 4.**
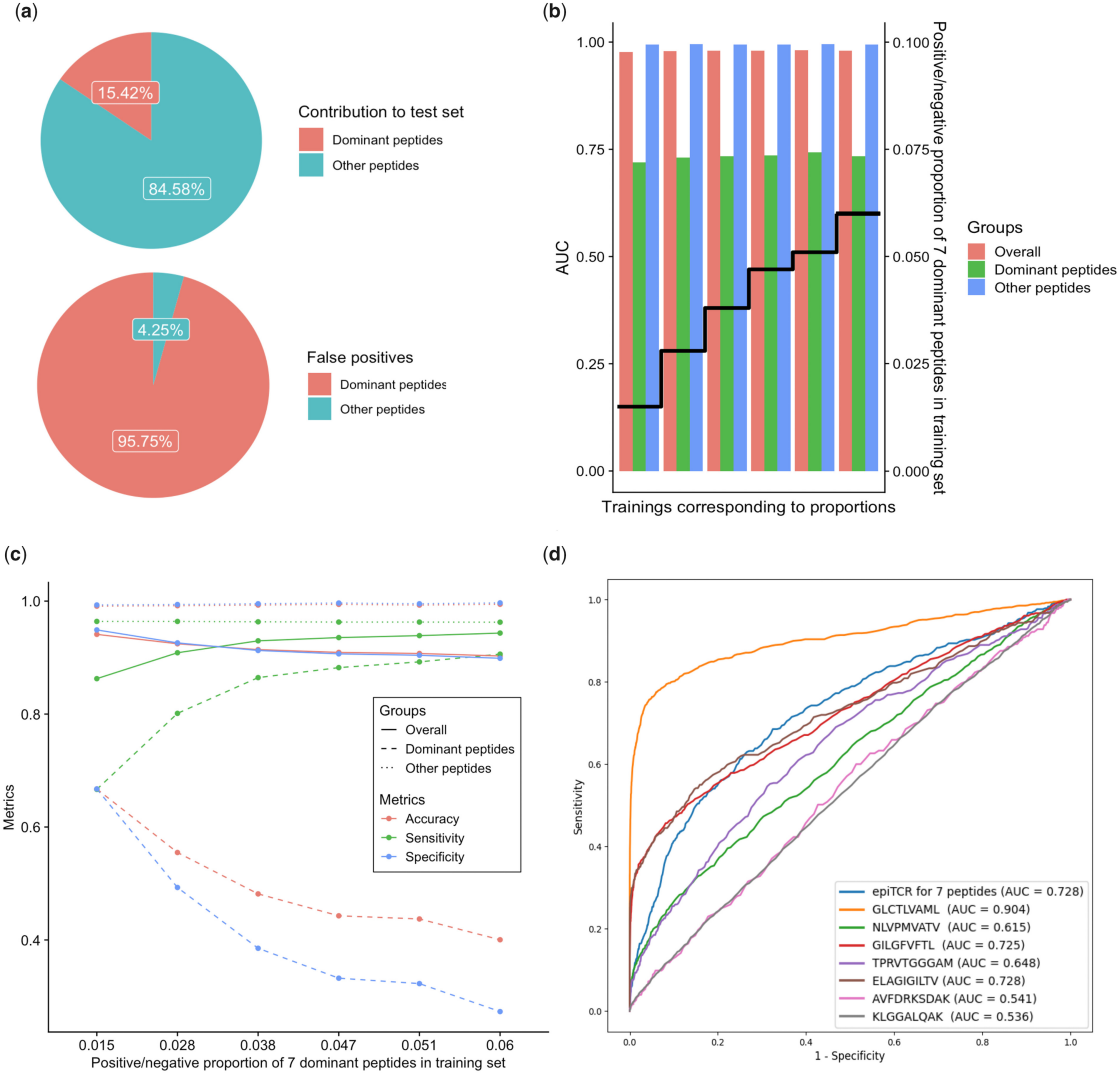
Impact of seven dominant peptides on binding prediction. (a) The dominant peptides contributed 15% of total binding pairs but made up 96% of false positives. (b, c) Models’ performance across different binding/non-binding proportions for dominant peptides in the training datasets, on three testing groups: overall, only dominant peptides, and other peptides. (d) Peptide-specific models also gave an unfavorable performance.

Dissecting the epiTCR specificity in the TCR–peptide benchmark, we discovered seven epitopes that appeared frequently in the training and test sets (so-called “dominant”) and contributed to the majority of false positive predictions (Fig. 4a). These dominant peptides are GLCTLVAML (GLC), NLVPMVATV (NLV), GILGFVFTL (GIL), TPRVTGGGAM (TPR), ELAGIGILTV (ELA), AVFDRKSDAK (AVF), and KLGGALQAK (KLG) (found in a total of 462 555 pairs) (Table S6). Figure 4a illustrates as a representative, the disproportion of false positives contributed by these seven peptides in the first test set of our TCR–peptide binding benchmark. Similar results for other test sets are also reported (Supplementary Table S4). Three among those seven peptides (GLC, NLV, and GIL) were also identified by NetTCR that they have dominant frequencies in the training set but were not linked to the model’s performance (Montemurro et al. 2021).

To search for a good model for predicting those dominant peptides binding, our first attempt was to train new models on five new training datasets with varying ratios of binding/non-binding data of the seven peptides, while the other peptides’ composition remained unchanged, derived from our original training dataset. The original training binding/non-binding ratio of the seven dominant peptides (at 0.051) and the same ratio of other training peptides (at 0.015) was also included in this evaluation. The models corresponding to those ratios were then evaluated on the same test set, i.e. test set numbered one in the TCR–peptide benchmark. The performance of the new models built on each new training dataset was calculated on three groups of data: overall (all peptides), only dominant peptides, and other peptides (Fig. 4b and c). Interestingly, the observed AUC on all peptides and on other peptides across models were stable and high, while the AUC on the dominant peptides was constantly low (Fig. 4b). Changes on the dominant peptide-related prediction were clearly observed on the other three performance metrics: accuracy, sensitivity, and specificity (Fig. 4c). While accuracy, sensitivity, and specificity on the other peptides remained constant, the respective metrics in the overall prediction followed the same trend as the performance of the dominant peptide classification. In other words, the seven peptides prediction dominated the performance of the overall prediction. With training sets of varying binding/non-binding ratios of dominant peptides, we could not find any optimal ratios that led to balanced and high sensitivity and specificity.

As the second attempt to improve the dominant peptide prediction as well as to ameliorate the overall prediction specificity, we tried to train distinct prediction models for those peptides, leading to a total of seven peptide-specific models and one model for all dominant peptides. However, we could not find any model that worked well on these peptides (Fig. 4d). Indeed, most newly trained models’ AUC ranged from 0.536 to 0.728 with a harsh trade-off between sensitivity and specificity. The GLC model was an exception and had an AUC of 0.9. However, its corresponding sensitivity and specificity remained relatively low. Overall, this result identified a group of peptides that appeared frequently in the data and challenged the TCR–peptide binding prediction.

### 3.4 epiTCR can scale to large datasets

**Figure 5.**
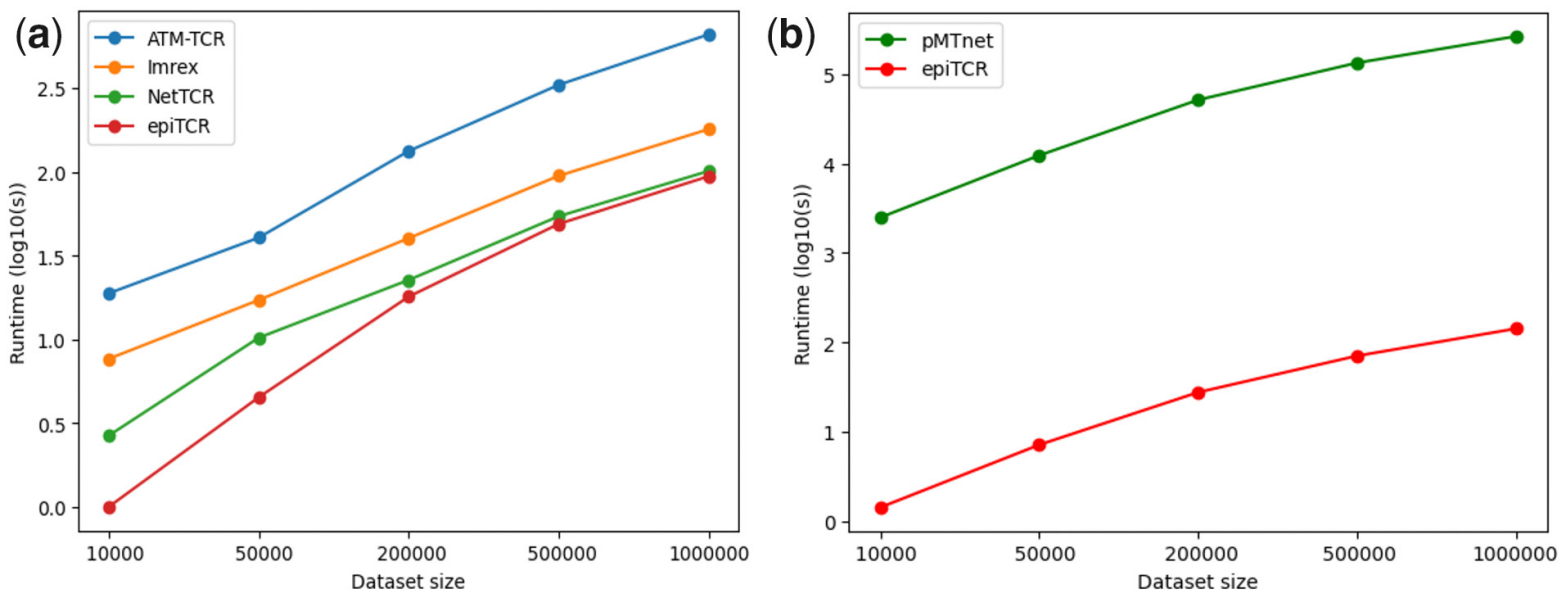
The runtime of ATM-TCR, Imrex, NetTCR, pMTnet, and epiTCR correspond to five randomly generated datasets. The benchmark is done in two cases: without MHC (left) and with MHC (right).

We evaluated the scalability of epiTCR on five datasets randomly generated with increasing sizes from 10 000 predicted pairs to 1 000 000 predicted pairs (Fig. 5). ATM-TCR and pMTnet need GPU to make predictions, so we ran those tools on machines having 755 GB RAM with 128 GPU cores. The other tools were run on the same amount of RAM and 128 CPU cores. As a result, all predictions, except pMTnet showed an acceptable running time. For the TCR–peptide interactions, epiTCR consumed approximately 94 s for the largest dataset, while all other tools also gave prediction within an hour. Predicting TCR–pMHC binding, epiTCR needed approximately 142 s to finish predicting the largest dataset, while pMTnet needs more than 3 days (around 73 hours) to solve the same request.

### 3.5 Application of epiTCR in the prediction of TCR-bound neoantigen

Recently, neoantigen-based cancer immunotherapies have demonstrated good clinical trial outcomes and attracted increasing research efforts (Peng et al. 2019; Zhang et al. 2021c). Therefore, we applied epiTCR model to predict the binding between TCR and tumor neoantigens (Supplementary Table S5). From our initial datasets, the neoantigens were retrieved by searching through five curated databases: TSNAdb (Wu et al. 2022, 2018), NeoPeptide (Zhou et al. 2019), dbPepNeo (Tan et al. 2020; Lu et al. 2022), NEPdb (Xia et al. 2021), and TANTIGEN (Olsen et al. 2017; Zhang et al. 2021a). All databases report antigens from published works and TSNAdb additionally includes the mutations found in The Cancer Genome Atlas (TCGA), IEDB (Vita et al. 2019), and The International Cancer Genome Consortium Data Portal (ICGC) (Zhang et al. 2019). The small overlapping neoantigens between cancer types were also found. The binding ground truth is from our initial pairing collection with a total of 57 989 non-redundant neoantigen-TCR pairs, in which 57 849 pairs were in the melanoma group, 31 pairs were in the breast cancer group, and 340 pairs were related to other cancer types (Supplementary Table S6).

**Figure 6.**
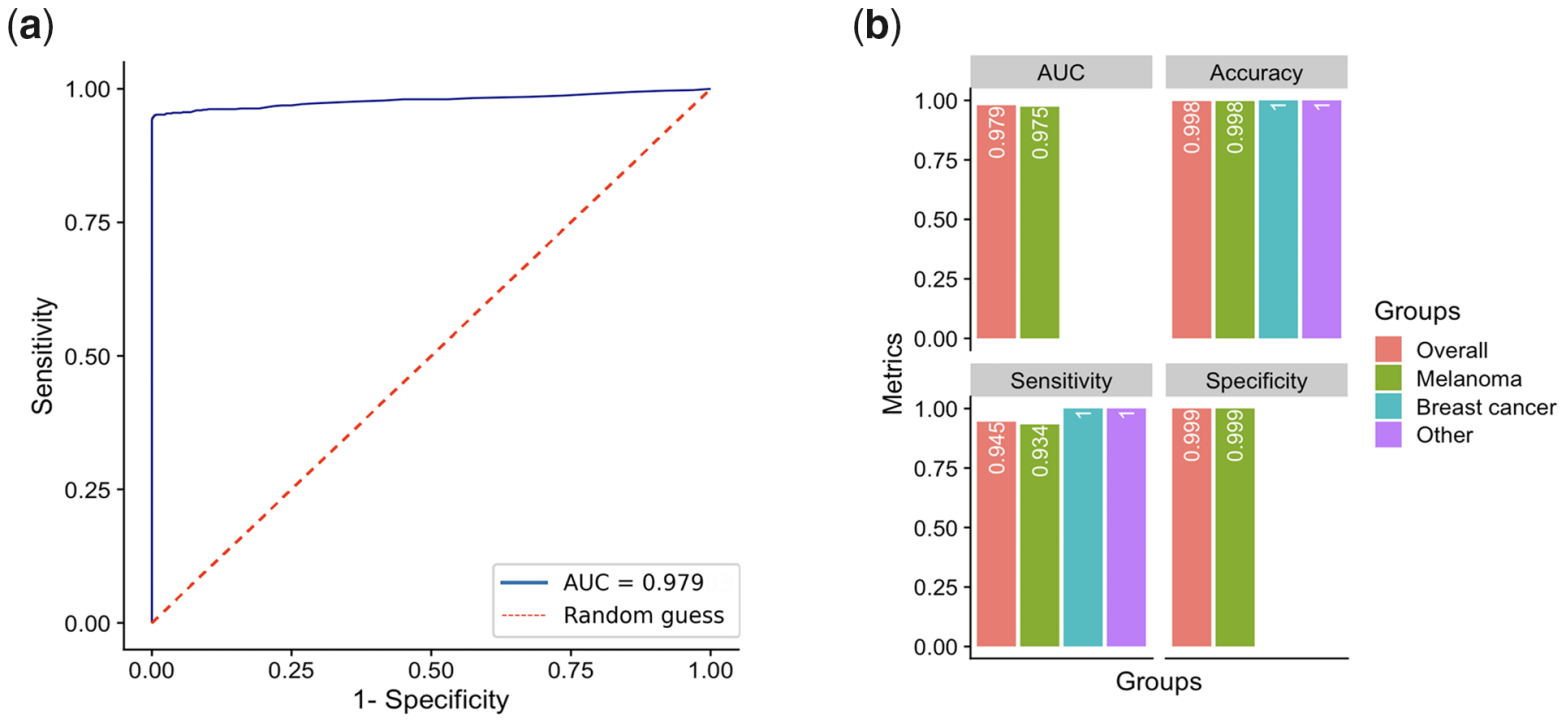
The performance of TCR-neoantigen binding prediction using epiTCR model on (a) all curated neoantigens as shown by ROC plot and (b) cancer-based categories at cutoff 0.5 (AUC, accuracy, sensitivity, and specificity). Blank columns indicated unavailable data due to zero non-binding observation in the indicated groups.

We calculated the epiTCR’s prediction performance on the neoantigen set as a whole and on cancer-based categories (Fig. 6). Overall, epiTCR model showed good prediction with AUC = 0.979 (Fig. 6a), a good sensitivity at 0.945, and high accuracy and specificity at the default probability cutoff of 0.5 (Fig. 6b). Neoantigen prediction for melanoma also showed good performance with AUC at 0.975, sensitivity at 0.934, and comparable accuracy and specificity with the overall prediction. Besides, neoantigen prediction in breast cancer and other cancers also showed high accuracy and sensitivity. However, due to the lack of non-binding data in those groups, the full performance assessment could not be completed.

### 3.6 Influence of peptide sequences on the prediction of TCR–peptide interaction

**Figure 7.**
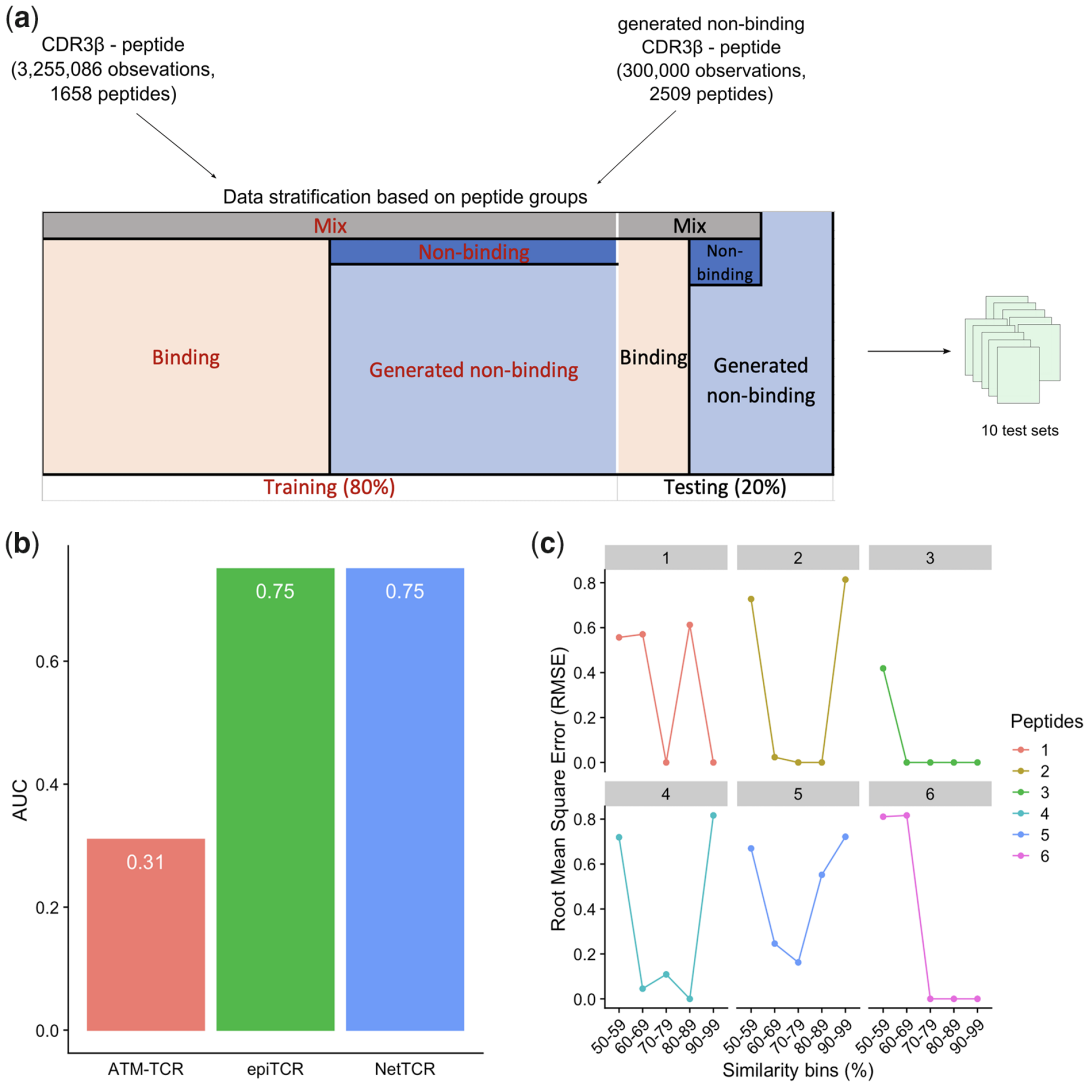
Prediction on observations derived from unseen peptides. (a) Data organization with the use of generated non-binding peptides to balance with the number of binding peptides in training set. (b) The prediction performance (mean AUC) of epiTCR, NetTCR, and ATM–TCR on interactions of unseen peptides from training. (c) Influence of peptide sequence similarity in training set on the predicted labels of peptides in the test set. Six peptides were chosen to represent six groups within the test sets and the proportion of their predicted labels were compared with the proportion of training labels at different levels of Levenshtein similarity using RMSE. The lower the RMSE, the more similar between predicted labels and training labels within a particular bin of peptides at the same level of sequence similarity.

Despite having collected >3 million TCR-peptide observations, the number of unique peptide sequences in our collection is relatively small (1658 peptide) compared to the number of TCR sequences. This suggests that the peptide sequences might have bigger influence on prediction outcomes when a model is trying to learn the TCR–peptide interaction pattern. To verify this, we first re-organized our data into training and test sets so that not only the observation pairs but also the peptides in these pairs in the test set did not overlap with those in the training set. We applied two criteria on the training set: (i) equal number of binding and non-binding peptides to provide balanced training data and (ii) maximize the number of peptides used for training to provide the most diverse patterns. However, the small number of unique peptides from non-binding pairs (7 peptides from 443 485 non-binding pairs) was not enough to match the number of peptides from binding pairs. To overcome this, we generated 300 000 non-binding pairs by randomly pairing 2509 wild-type peptides with 5776 known TCR, assuming that wild-type peptides will not bind to TCR and elicit T-cell responses (Supplementary Methods). As a result, 80% peptides were used in the training set, comprising of mix peptides (peptides that were found in both binding and non-binding pairs) binding peptides, non-binding peptides and generated non-binding peptides (Fig. 7a; Supplementary Methods). The remaining peptides and their related observations were used to generate 10 different test sets. Next, NetTCR, ATM-TCR, and epiTCR were re-trained on this new training set and then evaluated on the ten new test sets. The results show that NetTCR and epiTCR could predict interactions of unseen peptides with the mean AUC of 0.75, while ATM-TCR failed to classify interactions between TCR and new peptides (mean AUC of 0.31) (Fig. 7b). The decrease in models’ performance when tested on TCR–peptide pairs of unseen peptides indicates that the peptide sequences had significant impact on the prediction outcomes.

Having demonstrated the influence of peptide sequences on model prediction, we next asked whether the prediction was based on peptide similarity (i.e. a peptide in test set which was highly similar to one in training set would receive the same label). To answer this question, we compared the prediction labels of TCR–peptide pairs from a peptide in the test set with the labels of TCR–peptide pairs from similar peptides in the training set. Six peptides, representing six distinct groups in the test set [clustered based on their pairwise Levenshtein similarity (Levenshtein 1966)], were chosen. For each representative peptide, we grouped the training peptides into bins based on their similarity with the representative and calculated the root mean square error (RMSE) between the proportion of labels (binding/non-binding/mix) within each training bin and their respective proportion of predicted labels of the representative peptide (Fig. 7c). Our results showed that there are indeed peptides (peptides 1, 3, and 6) which shared similar predicted labels (low RMSE) to their corresponding bins of highly similar peptides in the training set. However, this trend was not conserved for all peptides (see different trends in peptides 2, 4, and 5), suggesting that it was not solely the peptide sequence similarity that dominated the learning space of ML models when learning TCR–peptide interactions.

## 4 Discussion

TCR–peptide binding prediction, which aims to identify epitopes capable of triggering T-cell responses, is a key step in the development of immunotherapy. Multiple TCR–peptide binding prediction tools have been published using a wide range of algorithms and datasets for training and validation (Dash et al. 2017; Gielis et al. 2018, 2019; Jokinen et al. 2021; Lin et al. 2021; Lu et al. 2021; Montemurro et al. 2021; Moris et al. 2021; Sidhom et al. 2021; Springer et al. 2020, 2021; Weber et al. 2021; Zhang et al. 2021b; Cai et al. 2022). In this work, we attempted to collect the most up to date TCR–peptide binding data from multiple publicly available sources and merged them into a unified dataset in order to build a prediction tool called epiTCR. Our benchmarks showed that most existing TCR–peptide binding prediction tools exhibit high classification specificity but low sensitivity. epiTCR was the only tool able to capture a large number of binding pairs while maintaining an acceptable specificity and scale well to large datasets.

A reason for the better performance of epiTCR would be the collection of data from multiple sources with diverse peptides and pathologies. The number of observations used for epiTCR was larger than the amount of data in any other published works [over 3 million observations compared to over 400,000 observations in NetTCR (Montemurro et al. 2021), and around 300 000 observations in ATM-TCR (Cai et al. 2022)]. This significantly larger dataset allowed for a training set with diverse peptide sequences and multiple non-overlapping testing sets to ensure a fair assessment of model performance. Indeed, when comparing the performance of multiple tools on these same testing sets, most tools did not perform well, or in the case of NetTCR, required re-training, suggesting that the dataset had a significant impact on performance, and in the task of TCR–peptide binding prediction more data are still required.

Given the severely imbalanced labeled data with non-binding interaction accounts for 30-folds more than binding data, the learning strategy may play a vital role in the model performance. In deep learning-based classification models such as NetTCR, ImRex, ATM-TCR, and pMTnet, the training set is uniformly split into batches with defined sizes (Lu et al. 2021; Montemurro et al. 2021; Moris et al. 2021). This helps the models learn all non-binding patterns from the non-binding data. In contrast, the training set for epiTCR was controlled at 1:10 binding: non-binding observation ratio, which helped it learn more binding patterns from the binding data. Therefore, the differences in training strategies are perhaps one of the main reasons for the outperformance of machine-learning models, epiTCR in particular, compared to deep learning models in the task of TCR–peptide binding prediction.

The data were also imbalanced in the proportion of input peptides. We identified seven dominant epitopes that heavily impact the learning model (Fig. 5). A small number of peptides have rare binding data and/or are unevenly distributed in either binding or non-binding observations. Consequently, the prediction performance in these cases cannot be evaluated properly. This represents an ongoing challenge of TCR–peptide binding prediction and could potentially be addressed by providing more data from assays such as single-cell sequencing.

TCR–peptide binding prediction is getting more attention because of its wide application in immunotherapy, particularly in personalized cancer treatment. A common workflow for neoantigen prediction relies on the somatic variant calling from patient DNA-seq and/or RNA-seq data to estimate the pMHC binding affinity, thereafter, identify the candidate neoantigens (Hundal et al. 2016). Some other works investigated ranking algorithms to increase the chance of identifying the “real” neoantigens (Gartner et al. 2021). However, *in vitro* experiments reveal the weakness in both approaches, which lies in the fact that only a very small number of predicted peptides were experimentally validated neoantigens (Garcia-Garijo et al. 2019). Therefore, the integration of the TCR–peptide binding prediction tool, particularly epiTCR, into known pipelines can open an innovative approach to filter candidate neoantigens. However, as more data are still desired for better prediction, more experimental validations of neoantigens are still needed for epiTCR to demonstrate its application in immunotherapy.

To the best of our knowledge, all TCR–peptide binding prediction tools, including epiTCR, use the same strategy of splitting training and test set in which the TCR–peptide pairs in the training are not repeated in the test set. However, little attention is paid to the fact that the peptide sequences in the training are in fact repeated in the test set and the number of unique peptide sequences are far less than the number of observations (1658 peptides versus >3 million observations), raising the question about the influence of peptide sequences on the prediction outcomes. To address this question, a different splitting strategy was needed in which both the peptide sequences and observation pairs in the training set were not seen in the test sets. The results indeed demonstrated a considerable influence of peptide sequences on the prediction outcomes, but this influence was different depending on the peptides (Fig. 7). The models built on this new training set (epiTCR and NetTCR) could still predict the interactions with modest performance (mean AUC of 0.75). The usefulness of this new splitting strategy and further improvement of prediction models trained on such strategy are important research questions for future studies. What is clear from this investigation is that future improvements of TCR–peptide binding prediction tools depend in part on more balanced data and more diverse peptide sequences.

In conclusion, we presented epiTCR, a simple ML prediction tool, that is among the most well-performing in all aspects thanks to the use of up-to-date binding data from public sources. It produces TCR–peptide prediction using the minimal required input, only CDR3β and peptide sequences. The use of epiTCR will contribute to the ongoing quest in identifying cancer neoantigens for the development of precision cancer immunotherapy.

## Supplementary Material

btad284_Supplementary_Data
