# Supplementary material for: epiTCR: a highly sensitive predictor for TCR–peptide binding
Source: Bioinformatics. 2023 Apr 24;39(5):btad284. doi: 10.1093/bioinformatics/btad284 (PMC10159657; doi:10.1093/bioinformatics/btad284)
Supplement: btad284_Supplementary_Data [file btad284_supplementary_data.zip › epiTCR-Supp-v2-major-revision.docx]

epiTCR: a highly sensitive predictor for TCR-peptide binding - Supplementary information

My-Diem Nguyen Pham^1^, Thanh-Nhan Nguyen^1^, Le Son Tran^1,2^, Que-Tran Bui Nguyen^1^, Thien-Phuc Hoang Nguyen^1^, Thi Mong Quynh Pham^1^, Hoai-Nghia Nguyen^2,3^, Hoa Giang^1,2^, Minh-Duy Phan^1,2^, Vy Nguyen^1^

^1^ Medical Genetics Institute, Ho Chi Minh City, Vietnam

^2^ NexCalibur Therapeutics, Delaware, USA

^3^ University of Medicine & Pharmacy, Ho Chi Minh City, Vietnam

Corresponding authors:

Vy Nguyen

nttv.2002@gmail.com

Minh-Duy Phan

pmduy@yahoo.com

# Supplementary methods

## Data collection

We gathered TCR – peptide binding binding data mainly from four public databases: the TBAdb, VDJdb, McPAS-TCR, and the Immune Epitope Database (IEDB). The TBAdb dataset was retrieved from the link: https://gitlab.com/immunomind/immunarch/-/blob/master/private/TBAdb.xlsx on June 16th, 2022. The VDJdb and IEDB dataset were both collected on June 16th, 2022. And the McPAS-TCR data were downloaded from the version updated on August 5th, 2021.

TCR – peptide non-binding data were retrieved from 10X Genomics Chromium Single Cell Immune Profiling, specifically from the project "Application Note - A New Way of Exploring Immunity" on June 20th, 2022. In this project, CD8+ T cells of four healthy donors were retrieved to test the binding with different pMHC complexes in dCODE^TM^ Dextramer reagents and binding T cells were then isolated by FACS. Although most CDR3β-pMHC combinations were non-binding, there were several binding combinations in this dataset, which were integrated into the binding dataset retrieved from the other four datasets as described above.

For HLA information, we searched the full sequence of MHC from the IMGT database on June 16^th^, 2022 (Table S2).

## Data preprocessing

Each dataset was first preprocessed individually. For CDR3β, amino acid sequences were used and where applicable (TBAdb, VDJdb, McPAS-TCR, and 10X datasets) the "C" starting and "F" ending characters in the CDR3β chain were removed similar to NetTCR[1]. In IEDB, due to the availability of curated and calculated CDR3β sequences[2]. Only one of them was used when they were exact matches. In cases where "C" and "F" had been partially removed in either the curated or calculated CDR3β chain, we choose to keep the shorter “C”, and “F” cleaved chains. All sequences containing unknown amino acids (encoded by X, O, special characters, and lowercase characters) were also removed. Empty peptides and unknown amino acids were also eliminated.

Redundant and label-conflicting TCR-peptide observations (both binding and non-binding interactions were recorded across different datasets) were removed for both binding and non-binding sets. All sequences were filtered by their length, with CDR3β length ranging from 8 – 19 amino acids, and peptide length from 8 – 11 amino acids. For datasets where HLA information was available, all HLA-I data were kept (to form CDR3β-pMHC data) whilst HLA-II were removed.

After data preprocessing, the final dataset has 3,255,086 CDR3β-epitope combinations (including 3,148,510 non-binding binding combinations) and 3,214,984 CDR3β-pMHC complexes (including 3,148,512 non-binding binding complexes). The code for data preprocessing is available on GitHub (https://github.com/nttvy/TCR-datasets).

## Annotation of pathogen sources and data validation methods

All collected TCR from VDJdb, TBAdb, McPAS-TCR, and 10X databases was from human. For data from IEDB, the TCR sources were from both human and mouse (based on the Host Name column in T cell essay metadata from IEDB) but the number of observations coming from mouse was very small (8 observations).

The pathogen source was indicated under different column names in different databases, specifically, column “Pathology”, “Organisms”, and “Epitope species” in McPAS-TCR, IEDB, and VDJdb, respectively. In TBAdb, this information was inferred from the “Disease name” and “Category” column. The detailed pathogen annotations were then converted into major groups based on their kingdoms (ie. virus, human, and other).

The annotation of validation methods to classify observations into “antigen-specific association” and “antigen-specific validated” group for the whole dataset was challenging. For IEDB, the interactions of curated TCR and peptides were assigned into “antigen-specific validated” group, while interactions of calculated TCR were categorized into “antigen-association validated” group. We also manually checked the validation method noted in the T cell assay metadata, including the columns for “Immunization comments”, “(validation) Method/Technique”, and “Assay Group”. For 10X, the data was clearly stated that TCR was identified through single-cell sequencing and the binding interactions were all confirmed by multimer assays. For the other three datasets, we classify the interactions as “antigen-specific validated” data if they were from multimer/tetramer experiments, ELISPOT, ELISA assays, targeted stimulation, or ex-vivo proliferation. The interactions from any other experiments were classified into the “antigen-specific association” group.

The detailed annotation of TCR-peptide pairs was provided in Supplementary File pathogen-source-categories.xlsx.

## Input feature encoding

Two types of data representation (one hot and BLOSUM62) were used in this project. For one hot encoding, each amino acid in the CDR3β peptide sequence and peptide sequence from the antigen was represented as one hot vector of 20 elements corresponding to 20 possible amino acids. The position for each amino acid type is fixed in the 20-element vector. i*th* element in the vector has one among two states: 1 if the i*th* amino acid is the same as the i*th* amino acids on the peptide sequence, else 0. Zero padding was then added to achieve fixed length representation (19 for CDR3b, 11 for epitope, and 34 for HLA). These resulting matrices were then flattened and finally concatenated, and the final vectors are provided for model training. The coding principle of BLOSUM62 was similar to one hot encoding, except that each amino acid in (any) peptide sequence was converted into the corresponding numerical vector defined by the matrix BLOSUM62[3]. Only BLOSUM encoding was used in the final version of epiTCR and mentioned in the main manuscript.


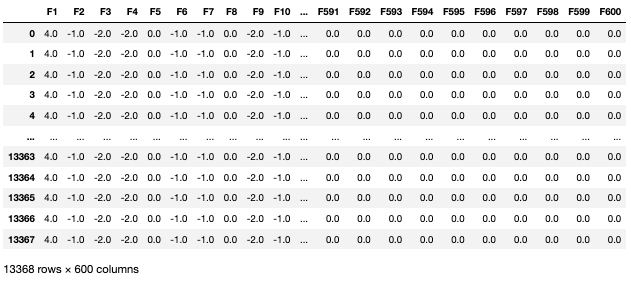


Figure S1 – Representation of input to Random Forest model for TCR-peptide classification. For each observation, the CDR3β and epitope were individually encoded using BLOSUM62 matrice, flattened, then concatenated. The resulting representation of the observation is a vector of 600 elements.

## Model training

To choose the most appropriate learning model for each data representation, we first used stratified five-fold cross-validation to train and validate different combinations between default machine learning models and data representation types. The machine learning options included SVM (with linear, polynomial, sigmoid, and RBF kernels), k-nearest neighbors, Random Forest, XGBoost, Linear Regression (with lbfgs, liblinear, sag, newton-cg solvers), and Gaussian Naive Bayes[4]. The tested data were BLOSUM62 encoded.

Because of the imbalance between classes in the training data, we use the downsampling technique and implement RandomUnderSampler() function from *imblearn* library[5] to retrieve balanced training data in every stratified cross-validation fold and in the training process.

The validation resulted in the outperformance of Random Forest using BLOSUM62 encoded data (Supplementary Table S2). Therefore, we applied grid search and stratified five-fold cross-validation to select the most appropriate hyperparameters for Random Forest. The hyperparameter space consists of *n_estimators*, *max_features*, *max_depth*, *min_samples_leaf, min_samples_split,* and *bootstrap*. The optimized models ended up with 1) *n_estimators=300, max_features=15, bootstrap=False*, and 2) *n_estimators=300, max_features=20, bootstrap=True* for TCR-peptide and TCR-pMHC models, respectively. Finally, the models with optimized hyperparameters were chosen based on the AUC (validation set, stratified cross-validation) and were fit again on the full training set. The trained models are used as default models of epiTCR for benchmarking with other binding prediction tools.

## General testing on models’ performance (AUC, accuracy, sensitivity, and specificity)

ImRex, ATM-TCR, NetTCR, and epiTCR gave predictions to testing sets containing CDR3β-peptide pairs, while pMTnet and epiTCR gave predictions to other datasets containing CDR3β-pMHC complexes. All tools were run with their default settings, except NetTCR and ATM-TCR were additionally retrained using the same training set as epiTCR’s models.

Most benchmarked tools produced predicting scores ranging from 0 to 1, and scores closer to 1 indicated the more reliable prediction, except pMTnet. This tool gave predicted binding ranks that the smaller values correspond to better predictions. Therefore, the AUC score and ROC curve for ImRex, ATM-TCR, NetTCR and epiTCR were calculated and drawn via the roc_auc_score() function in scikit-learn. The AUC score and the ROC curve for pMTnet, on the other hand, were provided by pMTnet itself. The other three metrics of benchmarks: accuracy, sensitivity, and specificity were calculated with a default prediction score cutoff at 0.5 for ImRex, ATM-TCR, NetTCR and epiTCR, and with pMTnet binding rank cutoff at 0.02, 0.05, and 0.1.

## Model training for seven dominant epitopes

We identified seven epitopes that occur frequently in the test sets (around 15%) but contribute up to 98% of the false binding prediction. Therefore, we tried to train models dedicated to only classifying this group of epitopes. To do this, different epitope-specific datasets were split from our data collection. For the seven epitopes, a total of eight small datasets (one dataset consisting of all seven epitopes) were produced. The training sets and test sets were divided from those eight small datasets respecting the proportion of 80% training and 20% testing. The model training was processed individually for every epitope. For each epitope, we trained one Random Forest-based model to specifically classify the corresponding binding pairs. We also trained one model to classify all mentioned epitopes. Using the same training process as for our general prediction models on all epitopes, we also applied five-fold cross-validation and down sampling to train and validate the models. The hyper-tunned models were fit again on their full training sets and tested on the remaining 20% data. The prediction AUC was calculated and compared with the validation AUC achieved by our overall model (on all peptides).

## Runtime estimation

We assessed the runtime on five large datasets ranging from 10,000 to 1,000,000 data points. These datasets were randomly selected from our data collection. The tools were benchmarked in their default predicting pipeline with the default prediction model. The benchmarked pipelines consist of data representation and prediction. For only NetTCR, the code for prediction was merged with the model training task. Therefore, we split the code into two parts: the training part, and the predicting part. The trained model was then supplied to the predicting part to separately calculate NetTCR running time. The prediction runtime was recorded as wall clock time. The exact moment of start and end were retrieved from the system time. The benchmarks were done on two Ubuntu machines with the same configuration: 755 GB RAM, 128 GPU cores, and 128 CPU cores.

## Evaluation of model performance on prediction of TCR bound neoantigens

Neoantigens used for epiTCR neoantigen prediction were first queried in five neoantigen/cancer-related antigen databases: TSNAdb, NeoPeptide, dbPepNeo, NEPdb, and TANTIGEN. These databases also contained multiple subsets leading to a total of 26 datasets. Particularly, TSNAdb contained one subset for each of the cancer types in bladder, brain, breast, cervix, colorectal, head and neck, kidney, liver, lung, ovary, pancreas, prostate, skin, stomach, thyroid, uterus, 16 cancers from TCGA, 7748 tumor samples from ICGC, and peptides validated in IEDB. dbPepNeo contained MHC-I high-confidence (HC) neoantigens, medium-confidence (MC) neoantigens, fusion neoantigens, and non-coding neoantigens. And each of NEPdb, NeoPeptide, and TANTIGEN contained one dataset.

All the mentioned databases also contain wild-type and commonly mutated peptides, so we first filtered out wild-type, overexpressed antigens, and shared tumor-specific antigens (clearly indicated in TANTIGEN and TSNAdb-IEDB). We did not accept C/T mutated peptides as neoantigens but defined them as tumor-associated antigens. Therefore, all peptides derived from C/T mutation recorded in TSNAdb and NeoPeptide were also removed. Additionally, our overview search showed that antigens listed in dbPepNeo without the corresponding wild-type sequences were also not neoantigen-derived sequences. For the rest of the sequences, we manually checked for neoantigen from published publications. The full list of used neoantigens was described in Table S5. These neoantigens were then used as the ground truth to evaluate epiTCR’s prediction.

## Non-binding data generation

For TCR-peptide data, non-binding data was generated by random coupling wild-type peptides with TCR. This strategy is based on the finding about non-binding selection in T cells that has been mentioned by Tran *et al.*,[6]. Indeed, matured TCR leaving from thymus should not bind to any wild-type peptides in healthy human’s body. Therefore, we applied this strategy by first retrieving 2509 wild-type peptides from three public databases (NEPdb, high confidence and medium confidence dbPepNeo, TSNAdb-IEDB). Then each of them was combined with 100-150 random TCR (TCR related to autoimmune was removed) from our collected datasets. All those generated pairs were all assigned to non-binding observations.

## Data preparation for unseen epitope training and testing

To provide data for model training and testing where the epitope sequences in testing set are unseen in training set, we applied two important criteria: (1) using equal number of binding and non-binding peptides in the training set to minimize the potential bias caused by the association of most peptides with either binding or non-binding status, (2) maximizing the amount of data used for training to give the model enough information to predict the unseen peptides. To satisfy these stringent prerequisites, the peptides were at first categorized into three groups: the “mix peptides” were found in both binding and non-binding TCR-peptide interactions, the “binding peptides” found only in binding interactions, and the “non-binding peptides” found only in non-binding interactions. From the 10X database where we got the majority of our non-binding pairs, only 7 peptides were found and categorized as non-bindings. In contrast, we found 1608 binding peptides from the other databases. Therefore, we had to generate more non-binding peptides to achieve equal numbers of binding and non-binding peptides as set out in criteria 1 (see Supplementary method, section Non-binding data generation). In total, our final dataset contained 87 mix peptides (2,750,187 observations), 1564 binding peptides (67,044 observations), and 2516 non-binding peptides (737,855 observations). The training set consisted of 1251 binding peptides (53,330 observations) and 70 mix peptides (78,086 observations) (approximate 80% of total binding and mix peptides), and 1251 non-binding peptides (53,330 observations). Consequently, the remaining 17 mix peptides (445,077 observations), 313 binding peptides (13,714 observations), and 1265 non-binding peptides (335,374 observations) were left for performance evaluation. We randomly sampled ten testing sets. In each testing set, the number of binding observations was one-tenth the number of non-binding observations. This dataset (both training and testing sets) is available on epiTCR GitHub repository (https://github.com/ddiem-ri-4D/epiTCR).

## Training epiTCR model for unseen epitope classification

Similar to the general epiTCR model classifying CDR3β-epitope binding, we kept the same set of hyper-parameters to train another epiTCR version for unseen epitope classification. In particular, the hyper-parameters used were: *n_estimators=300, max_features=15, bootstrap=False.*

## Sequence similarity

The sequence similarity was calculated separately for epitope and MHC pseudo sequences. For each group, the non-redundant sequences were first gathered. The Levenshtein distance calculation is prepared via a python package *thefuzz*[7].

## The relationship between prediction outcomes of unseen peptides and labels of training peptides

epiTCR has proved to be able to classify the interactions of unseen peptides. It would be interesting to know if the interactions on seen peptides affected the prediction on new peptides. Particularly, the model might have put the same interaction labels on highly similar peptides. Therefore, this analysis was to identify if there were peptides whom prediction relies on the labels of any seen and similar individuals.

The pairwise similarity between peptides was measured using Levenshtein score. The ensemble of pairwise similarity between one peptides and other peptides formed the set of features of the considered peptide. To have a broad analysis on the peptides, we split the tested individuals into six distinct clusters using the squared Euclidean distance. In each cluster, we chose one epitope as the cluster representer. For each of those representing peptides, we calculated its similarity to all training peptides, then grouped the training peptides into bins based on the similarity scores. Within each bin, we calculated the relative amount of training data based on epitope label categories (mix, binding, and non-binding). Therefore, we have three features for each bin. We also calculated the ratio of predicted binding and non-binding interactions for the tested representers, hence forming a feature vector for each peptide. The difference between the peptide feature vector and the bin feature vector reflected the reverse correlation between the prediction and the training information respecting the bin similarity. If the prediction on unseen peptides was based on the similar training peptides, the difference between vectors is getting smaller. We evaluated such difference by calculating the RMSE between the bin feature vector and the peptide of consideration.

# Supplementary data

## Data preprocessing


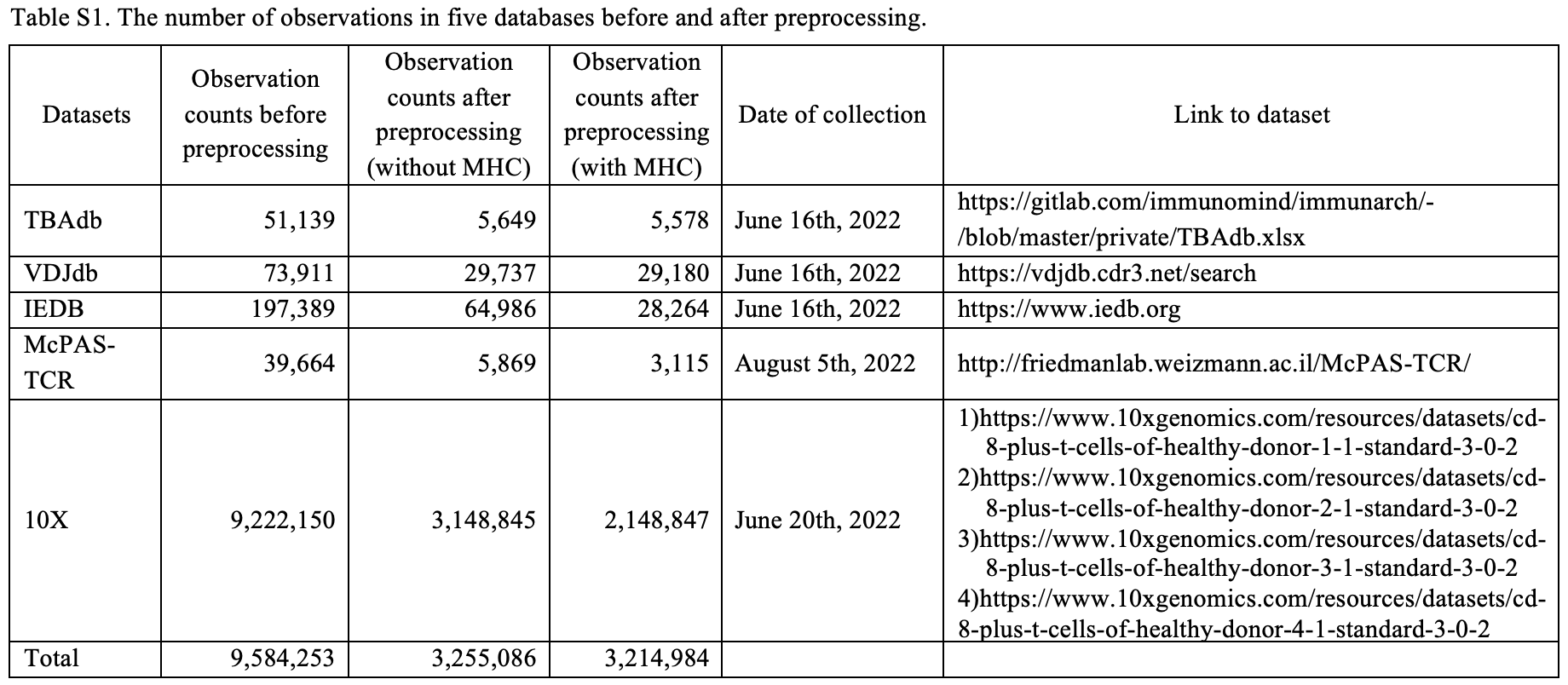


## Random Forest was the model of choice for prediction of TCR-peptide interactions


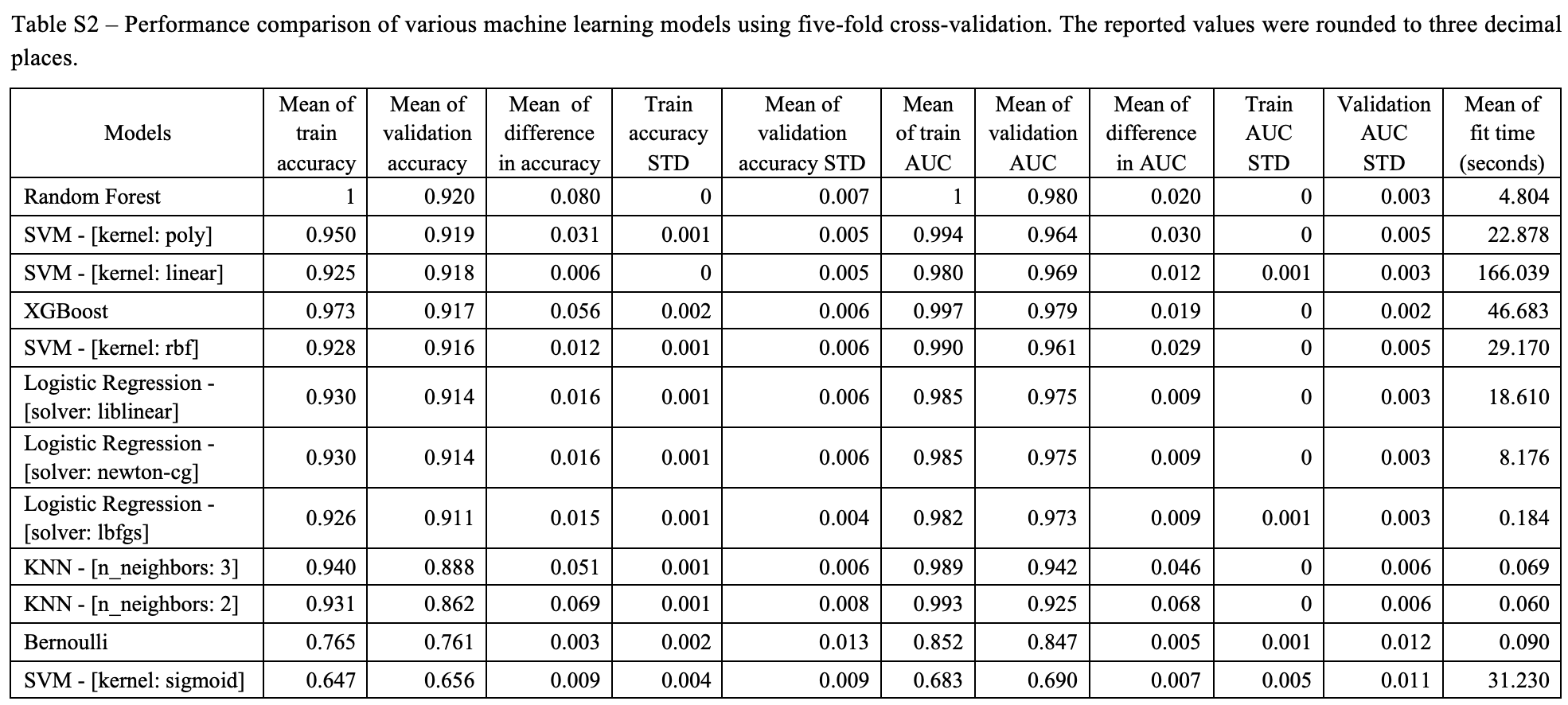


## Performance of NetTCR, ImRex, ATM-TCR, and epiTCR in predicting TCR-peptide binding


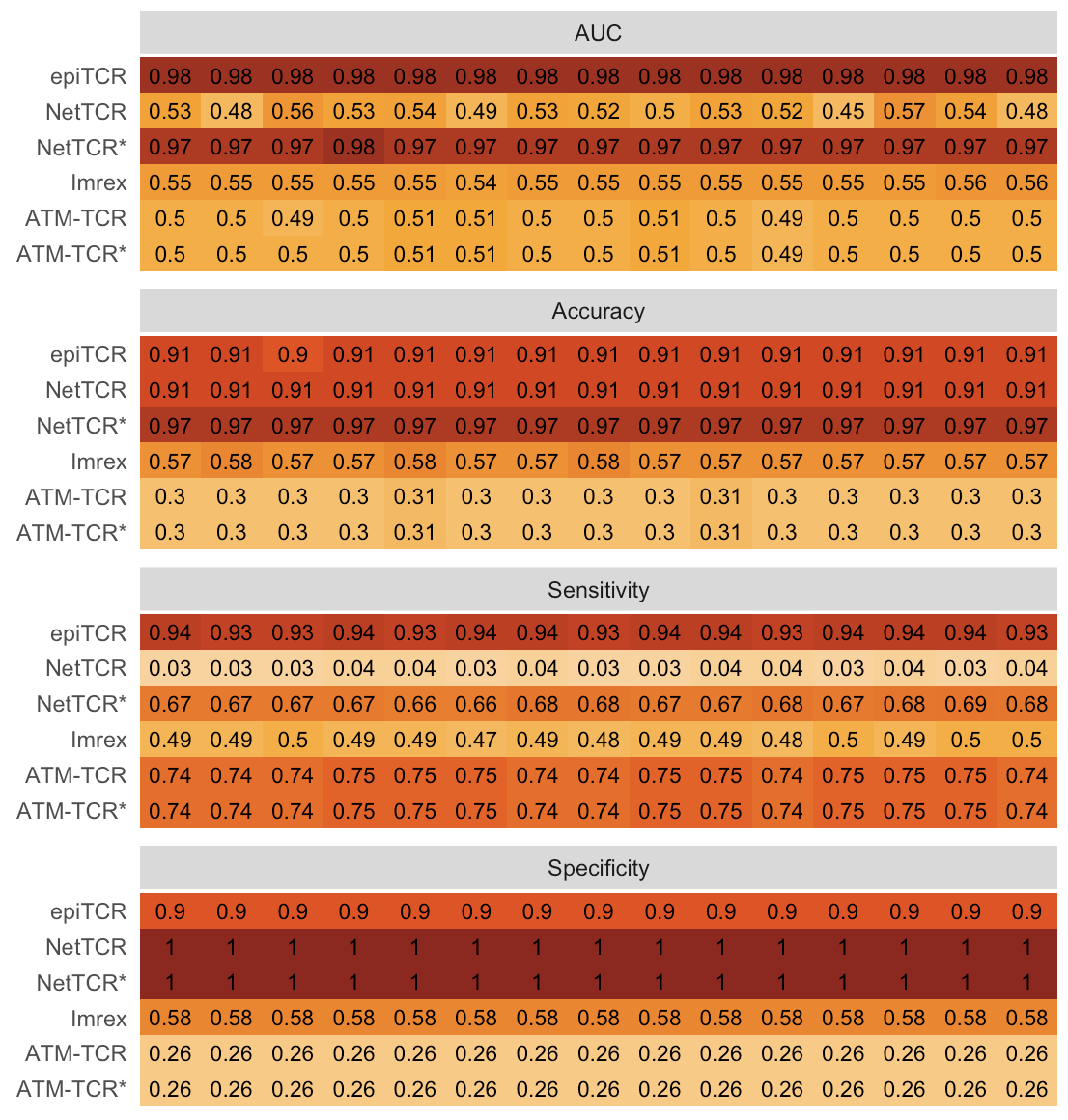


Figure S2 – The performance of epiTCR, NetTCR, Imrex, and ATM-TCR on AUC, accuracy, sensitivity, and specificity. An asterisk (*) after the tool's name indicates the retrained models. The darker shades show a better performance.


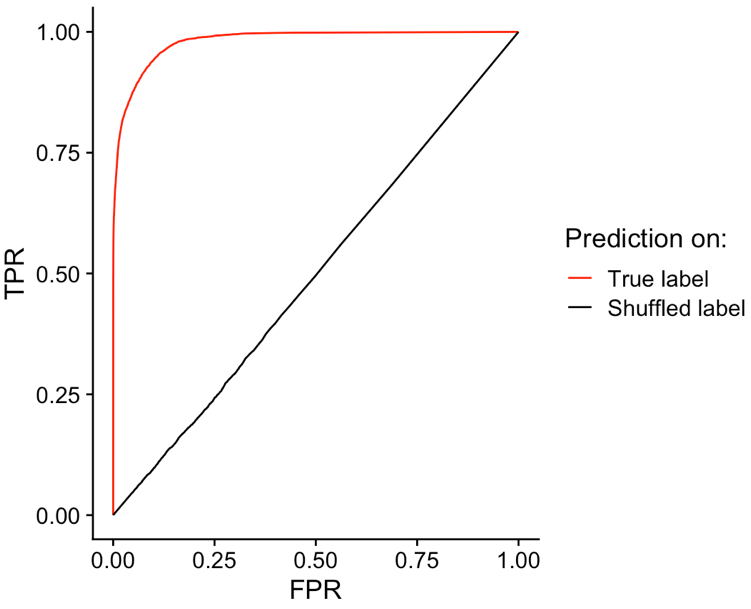


Figure S3 – The performance of epiTCR on the first test set and on the shuffled version of the first test set. The label of the first test set was randomly permuted, hence binding pairs become non-binding pairs. epiTCR prediction AUC on shuffled data was around 0.5, similar to random guess. This result proves that there is significant difference between the prediction on real data versus the shuffled data.

## Performance of epiTCR across different data categories


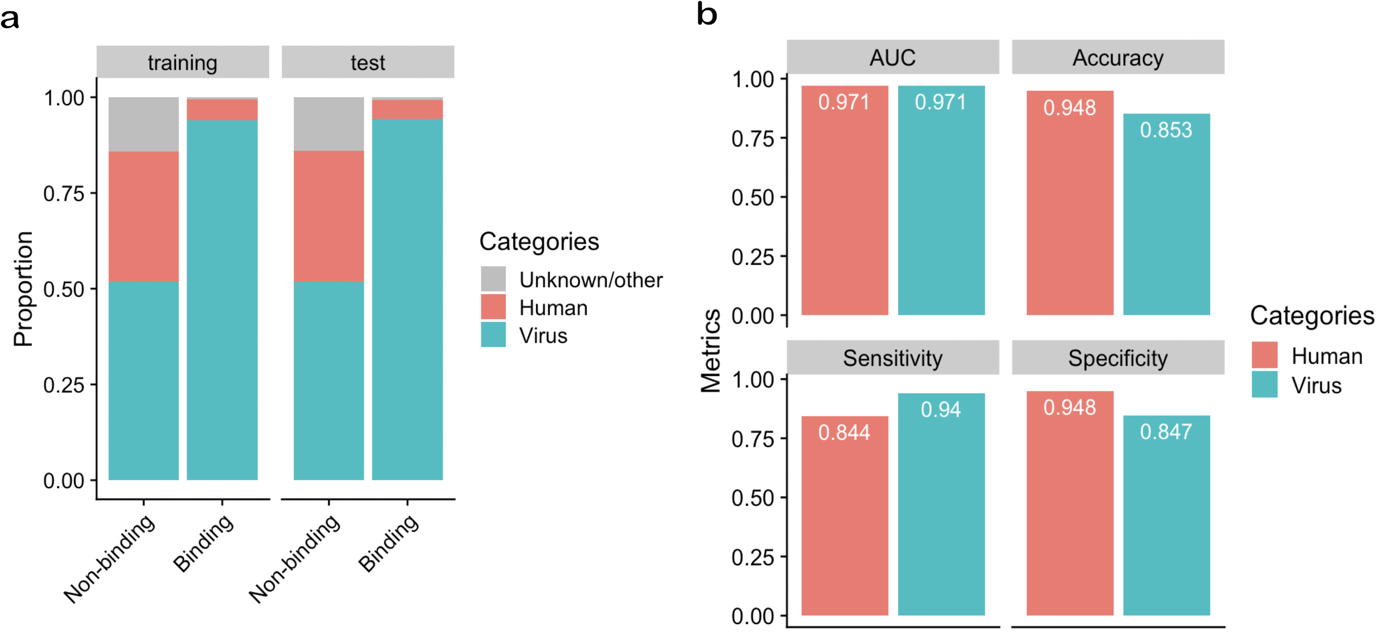


Figure S4 – The performance of epiTCR across different pathogen sources: human and virus. epiTCR AUC was relatively the same for the two data categories, but there was a trade-off between the sensitivity and specificity. Particularly, prediction on human TCR-peptide pairs had high specificity traded by the sensitivity, while the prediction on virus data pairs had high sensitivity but low specificity.


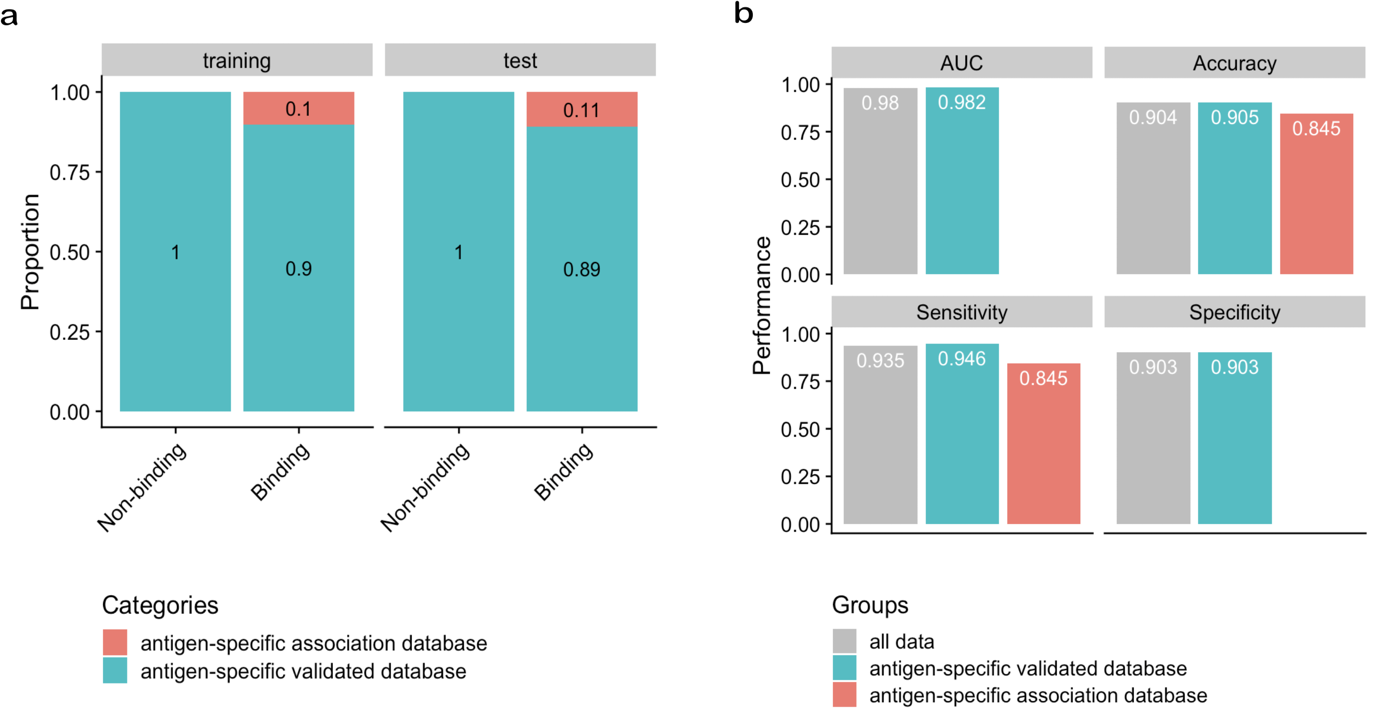


Figure S5 – The performance of epiTCR stratified by TCR categories. The proportion of antigen-specific validated data and antigen-specific association data in training set and test set, for binding and non-binding interactions (a), and the model performance on all data and on two data categories (b). Antigen-specific association database contained the TCR-epitope pairs that have not been validated or are unknown/unclear to have been validated. No non-binding data for antigen-specific association database was recorded. Therefore, the AUC and specificity on antigen-specific assocation was not calculated. The equal prediction performance on all data and on antigen-specific validated data suggested that there was almost no affect caused by the antigen-specific association data.

Table S3 – Number of observations of two databases in the training set and test set, for binding and non-binding data.

| Database | Training set | | Test sets | |
| --- | --- | --- | --- | --- |
|  | Binding | Non-binding | Binding | Non-binding |
| antigen-specific association database | 685 | 0 | 10815 | 0 |
| antigen-specific validated database | 5999 | 314681 | 89077 | 2833829 |

## Performance of epiTCR and pMTnet in predicting TCR-pMHC binding


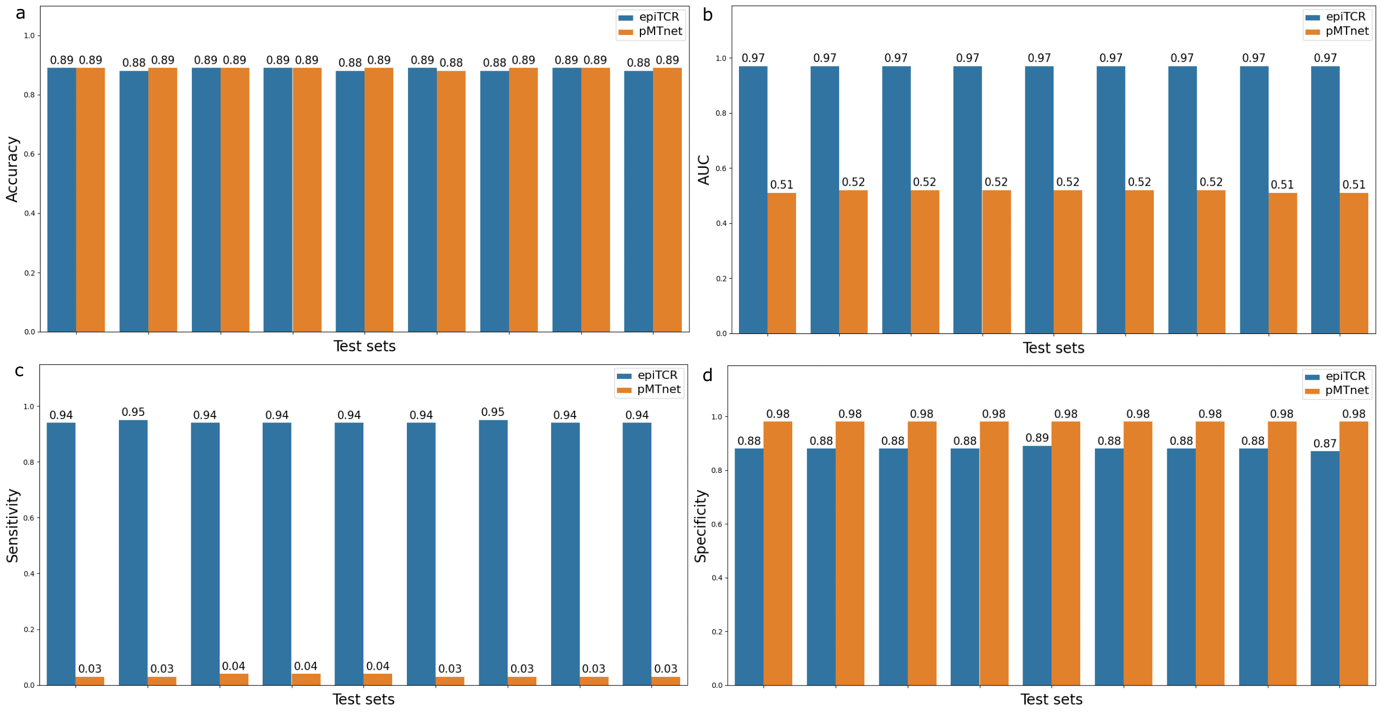


Figure S6 – Performance of epiTCR and pMTnet in TCR-pMHC binding prediction. Performance of pMTnet is calculated with binding ranking cutoff at 0.02.


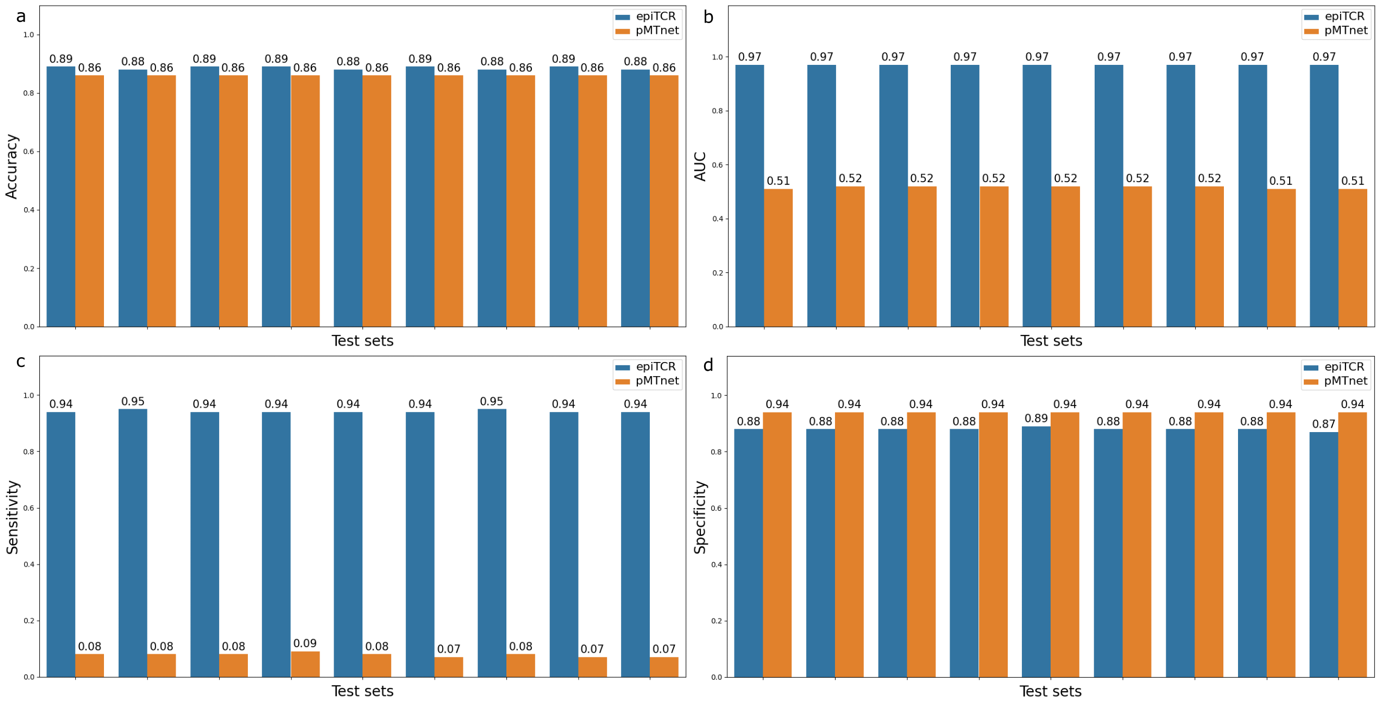


Figure S7 – Performance of epiTCR and pMTnet in TCR-pMHC binding prediction. Performance of pMTnet is calculated with binding ranking cutoff at 0.05.


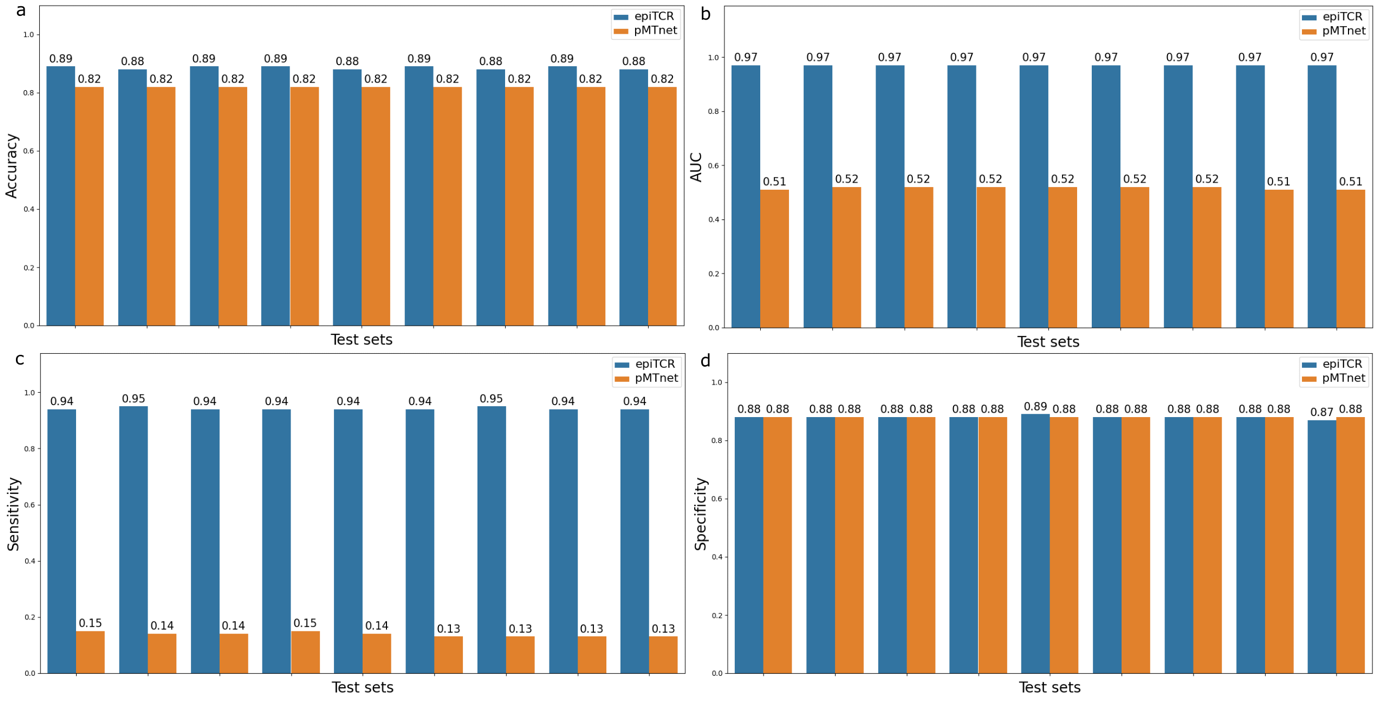


Figure S8 – Performance of epiTCR and pMTnet in TCR-pMHC binding prediction. The performance of pMTnet is calculated with the cutoff of 0.1.

## epiTCR revealed seven epitopes challenging for binding prediction


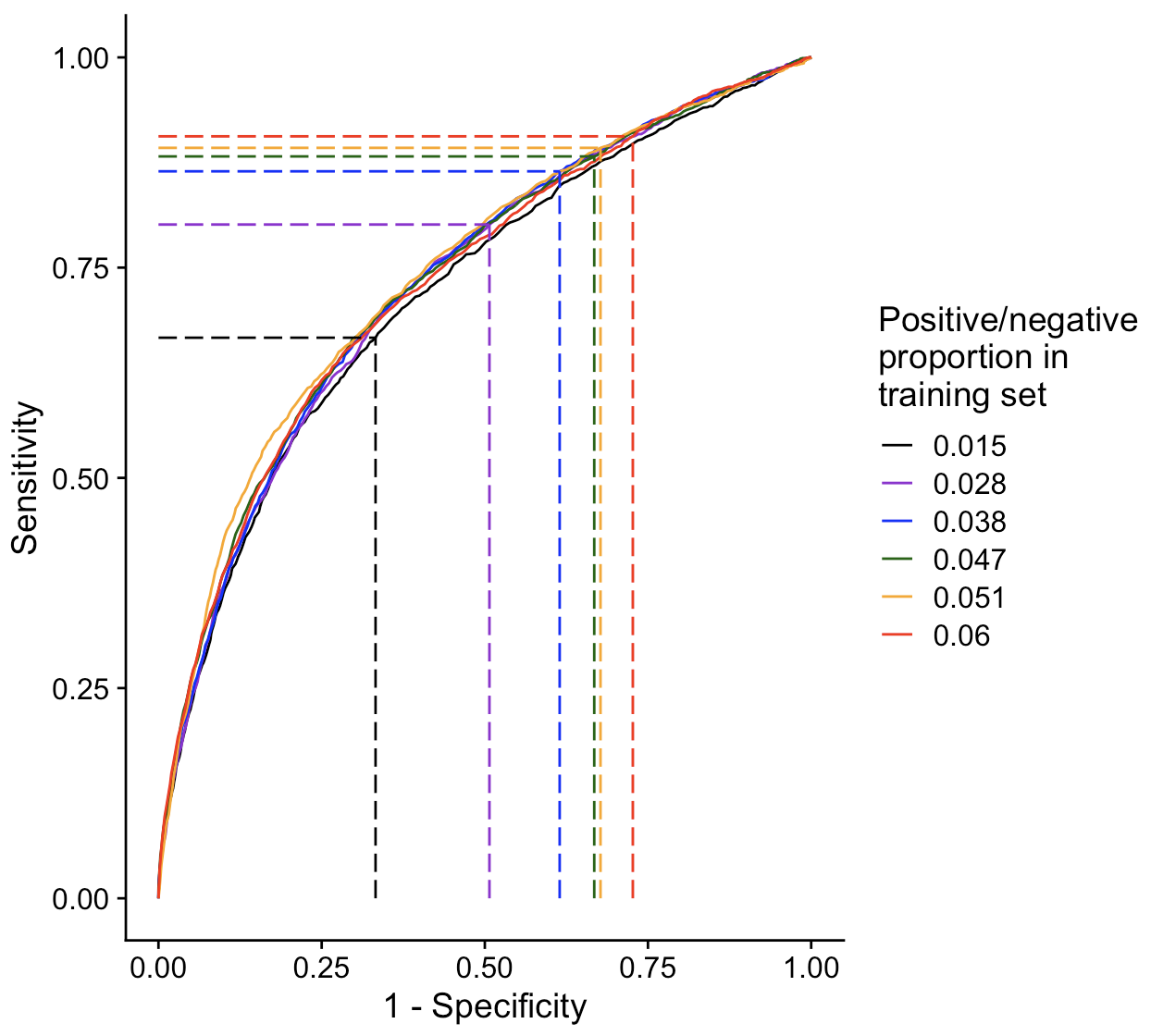


Figure S9 – ROC curves indicate the prediction performance corresponding to models trained on different training sets. Those training sets differed by the binding/non-binding proportion of participating CDR3β-peptide related to seven dominant peptides. Dashes indicate the corresponding True Positive Rate (Sensitivity) and False Positive Rate (1 – Specificity) at the default prediction probability cutoff of 0.5.

Table S4 – The proportion of dominant peptides in 15 test sets and the corresponding proportion of false positives contributed by those peptides.

| Test sets | Dominant peptides: GLC, NLV, GIL, TPR, ELA, AVF, KLG | |
| --- | --- | --- |
|  | Proportion of data in test sets (%) | Proportion of related false positives in test sets (%) |
| 01 | 15.42 | 95.75 |
| 02 | 15.27 | 95.35 |
| 03 | 15.44 | 95.79 |
| 04 | 15.56 | 95.86 |
| 05 | 15.36 | 95.66 |
| 06 | 15.52 | 95.28 |
| 07 | 15.29 | 95.36 |
| 08 | 15.34 | 95.35 |
| 09 | 15.48 | 95.32 |
| 10 | 15.40 | 95.89 |
| 11 | 15.34 | 94.84 |
| 12 | 15.44 | 95.45 |
| 13 | 15.32 | 95.56 |
| 14 | 15.18 | 95.74 |
| 15 | 15.36 | 95.74 |


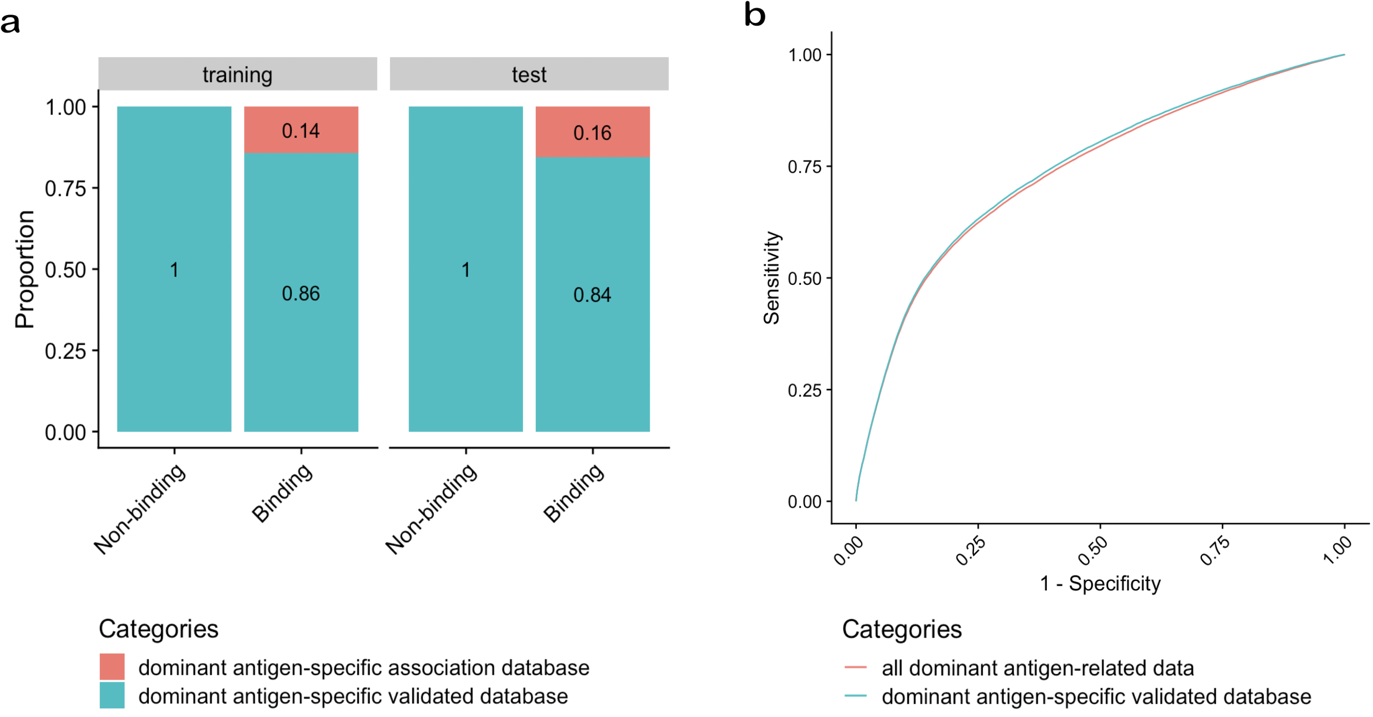


Figure S10 – epiTCR’s performance on different groups of dominant peptides. The proportion of dominant epitope-related observations in antigen-specific association database and in antigen-specific validated database, across training set and test set, for binding and non-binding data (a), and the performance of epiTCR on dominant epitope-related observations in all test population and in antigen-specific validated database (with AUC = 0.7377 and AUC = 0.7439, respectively) (b). Antigen-specific association database contains the TCR-epitope pairs that have not been validated or are unknown/unclear to have been validated. epiTCR performance on antigen-specific validated data was relatively the same as the prediction performance of epiTCR on all dominant epitope-based observations (Figure 4b), which suggests no significant impact by the unvalidated data on the prediction performance on dominant epitopes observations.

Table S5 – Number of dominant-epitope-related observations in two validation databases in the training set and test set, for binding and non-binding data.

| Database | Training set | | Test sets | |
| --- | --- | --- | --- | --- |
|  | Binding | Non-binding | Binding | Non-binding |
| antigen-specific association database | 362 | 0 | 5281 | 0 |
| antigen-specific validated database | 1940 | 42568 | 28701 | 383739 |


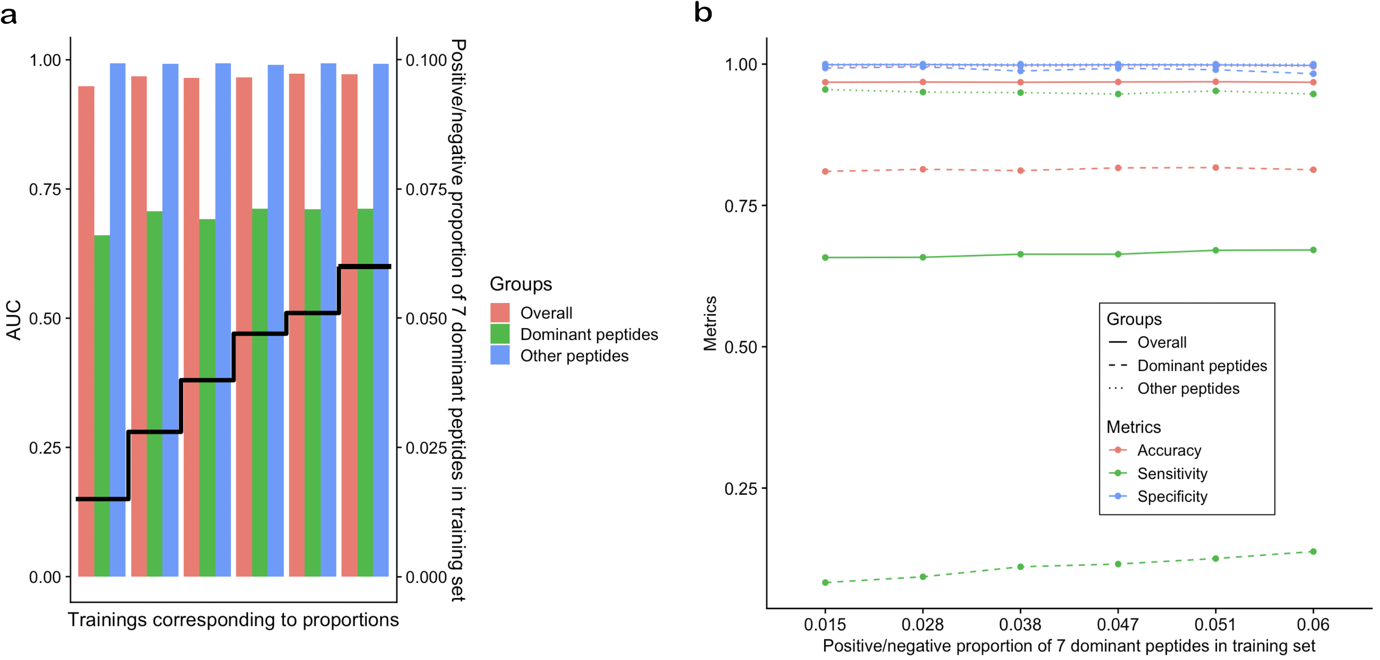


Figure S11 – NetTCR performance across different binding/non-binding proportions for dominant peptides in the training datasets, on three testing groups: overall, only dominant peptides, and other peptides. (a) AUC. (b) Accuracy, sensitivity, and specificity. This result shows that NetTCR also has difficulties in classifying interactions related to the seven dominant peptides, despite the amount of training data.


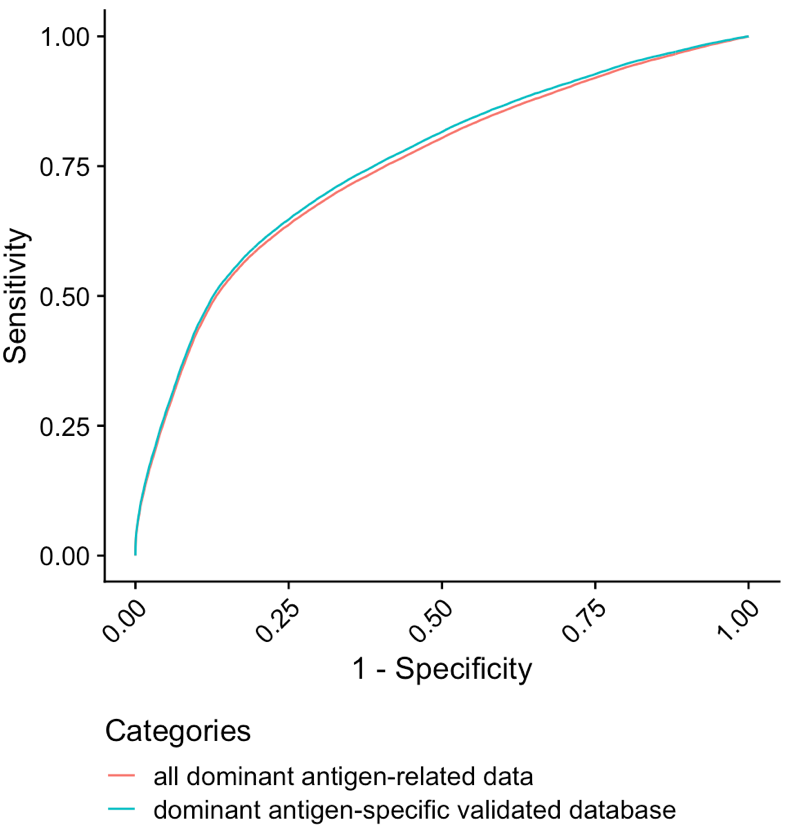


Figure S12 – epiTCR was re-trained on validated dominant epitope-related observations, then evaluated on all dominant epitope-related data and validated dominant epitope-related data. Although having been trained on validated data, the model still had difficulties in classifying dominant epitope interactions.

Table S6. Seven-epitope-related pair counts

| Epitope | Total counts |
| --- | --- |
| AVFDRKSDAK | 63,157 |
| ELAGIGILTV | 64,893 |
| GILGFVFTL | 67,505 |
| GLCTLVAML | 69,827 |
| KLGGALQAK | 63,241 |
| NLVPMVATV | 68,209 |
| TPRVTGGGAM | 65,723 |
| Total | 462,555 |

## Neoantigen prediction

Table S7. List of neoantigen used for epiTCR prediction (in a separated file).

Table S8. List of neoantigen-TCR pairs predicted by epiTCR corresponding to different groups of cancers (in a separated file).

# References

1. Montemurro A, Schuster V, Povlsen HR, Bentzen AK, Jurtz V, Chronister WD, et al. NetTCR-2.0 enables accurate prediction of TCR-peptide binding by using paired TCRα and β sequence data. Commun Biol. 2021;4: 1060.

2. Mahajan S, Vita R, Shackelford D, Lane J, Schulten V, Zarebski L, et al. Epitope specific antibodies and T cell receptors in the Immune Epitope Database. Front Immunol. 2018;9: 2688.

3. Henikoff S, Henikoff JG. Amino acid substitution matrices from protein blocks. Proc Natl Acad Sci U S A. 1992;89: 10915–10919.

4. Pedregosa F, Varoquaux G, Gramfort A, Michel V, Thirion B, Grisel O, et al. Scikit-learn: Machine Learning in Python. arXiv [cs.LG]. 2012. Available: http://arxiv.org/abs/1201.0490

5. Lemaitre G, Nogueira F, Aridas CK. Imbalanced-learn: A python toolbox to tackle the curse of imbalanced datasets in machine learning. arXiv [cs.LG]. 2016. Available: http://arxiv.org/abs/1609.06570

6. Tran NH, Li M. Predicting immunogenicity by modeling the central tolerance of CD8+ T cells in individual patients. bioRxiv. 2022. doi:10.1101/2022.07.05.497667

7. Thefuzz. Github; Available: https://github.com/seatgeek/thefuzz
